# VEGF regulates local inhibitory complement proteins in the eye and kidney

**DOI:** 10.1172/JCI86418

**Published:** 2016-12-05

**Authors:** Lindsay S. Keir, Rachel Firth, Lyndsey Aponik, Daniel Feitelberg, Susumu Sakimoto, Edith Aguilar, Gavin I. Welsh, Anna Richards, Yoshihiko Usui, Simon C. Satchell, Valeryia Kuzmuk, Richard J. Coward, Jonathan Goult, Katherine R. Bull, Ruchi Sharma, Kapil Bharti, Peter D. Westenskow, Iacovos P. Michael, Moin A. Saleem, Martin Friedlander

**Affiliations:** 1 Department of Cell and Molecular Biology, The Scripps Research Institute, La Jolla, California, USA.; 2 Academic Renal Unit, School of Clinical Sciences, University of Bristol, Bristol, United Kingdom.; 3 Queens Medical Research Institute, University of Edinburgh, Edinburgh, United Kingdom.; 4 Tokyo Medical University Hospital, Tokyo, Japan.; 5 Centre for Cellular and Molecular Physiology, University of Oxford, United Kingdom.; 6 National Eye Institute, NIH, Bethesda, Maryland, USA.; 7 The Lowy Medical Research Institute, La Jolla, California, USA.; 8 École Polytechnique Fédérale de Lausanne, Lausanne, Switzerland.

## Abstract

Outer retinal and renal glomerular functions rely on specialized vasculature maintained by VEGF that is produced by neighboring epithelial cells, the retinal pigment epithelium (RPE) and podocytes, respectively. Dysregulation of RPE- and podocyte-derived VEGF is associated with neovascularization in wet age-related macular degeneration (ARMD), choriocapillaris degeneration, and glomerular thrombotic microangiopathy (TMA). Since complement activation and genetic variants in inhibitory complement factor H (CFH) are also features of both ARMD and TMA, we hypothesized that VEGF and CFH interact. Here, we demonstrated that VEGF inhibition decreases local CFH and other complement regulators in the eye and kidney through reduced VEGFR2/PKC-α/CREB signaling. Patient podocytes and RPE cells carrying disease-associated CFH genetic variants had more alternative complement pathway deposits than controls. These deposits were increased by VEGF antagonism, a common wet ARMD treatment, suggesting that VEGF inhibition could reduce cellular complement regulatory capacity. VEGF antagonism also increased markers of endothelial cell activation, which was partially reduced by genetic complement inhibition. Together, these results suggest that VEGF protects the retinal and glomerular microvasculature, not only through VEGFR2-mediated vasculotrophism, but also through modulation of local complement proteins that could protect against complement-mediated damage. Though further study is warranted, these findings could be relevant for patients receiving VEGF antagonists.

## Introduction

Age-related macular degeneration (ARMD), the leading cause of vision loss in industrialized nations (1), affects 30 to 50 million people worldwide, but this is predicted to rise to 288 million by 2040 (2). There are 2 forms of ARMD, neovascular (wet) and atrophic (dry). Both show changes in the outer retina and can coexist in the same eye. Normally, retinal pigment epithelial (RPE) cells secrete VEGF, which has autocrine trophic effects (3), supports photoreceptors and, after crossing Bruch’s membrane, maintains the extraretinal vasculature of the fenestrated choriocapillaris (4). In dry ARMD, there can be subretinal deposits called drusen, photoreceptor dysfunction, RPE atrophy, and choriocapillaris degeneration, together called geographic atrophy (GA) (5). There is no treatment for GA. Wet ARMD is characterized by drusen, choroidal neovascularization (CNV), and retinal edema (1). High concentrations of VEGF contribute to CNV development (6), so wet ARMD is treated with intravitreal anti-VEGF injections. This therapy revolutionized ARMD patient care. While it does not reverse CNV, it does decrease macular edema that leads to reduced visual acuity. However, not all patients respond equally. Over 40% have stable or improved visual acuity (7), but 10%–30% of patients treated develop reduced visual acuity with regular repeated injections over time (8, 9). This could be due to the loss of VEGF’s trophic effects (4, 10). Mice develop choriocapillaris degeneration and photoreceptor dysfunction 3 days after genetic ablation of RPE-derived VEGF (4), while primates given intravitreal VEGF antagonists showed reduced thickness and number of fenestrations of the choriocapillaris maximal 4 days after treatment (11–13). This recovered 2 weeks later. Furthermore, cell culture studies suggested anti-VEGF agents cause RPE dedifferentiation (14), reduced barrier function (15), permeability (16), and impaired phagocytosis (17), but have no effect on apoptosis (18). Therefore, complete VEGF inhibition may be detrimental, but given the variability in reported effects, modifying factors could influence patient response and risk of developing side effects. A recent meta-analysis combining 13 studies reported reduced response to anti-VEGF therapy in patients homozygous for the complement factor H (CFH) polymorphism Y402H (19). The reason why these patients respond less well is unclear, but could suggest a relationship between VEGF and complement. This is supported by reports that choriocapillaris endothelial cell loss is an early feature of ARMD (20, 21) and that this is associated with increased deposition of complement membrane attack complexes (MACs) (21, 22).

Complement activation is evident in both types of ARMD, including GA, with complement deposits detected in drusen, on RPE cells, Bruch’s membrane, and the choriocapillaris (23, 24). The complement system is composed of over 30 proteins and can be activated by 3 pathways: the classical, lectin, and alternative pathways (25). Each pathway results in the formation of a C3 convertase, which hydrolyses C3 to C3a and C3b, and a C5 convertase, which cleaves C5 to C5a and C5b. C5 combines with C6-9, forming the MAC (25). Cells express inhibitory proteins that prevent inappropriate complement activation and cellular damage. Inhibitors can be membrane bound, like CD59, CD55, CD46 and Crry in mice, or circulate like CFH, which functions in serum or at the cell surface to stop alternative pathway activation (25). RPE cells synthesize CFH (26). From 30% to 50% of ARMD patients carry a CFH polymorphism (Y402H) (1, 27) that increases the risk of developing ARMD (1) and may predispose to complement activation (24). It is not fully understood how this contributes to ARMD pathogenesis, but suggests that complement regulation is important for the outer retina.

Another organ where VEGF and complement regulation are important is the kidney. The glomerular functional unit parallels that of the outer retina. The epithelial podocyte, like the RPE cell, produces VEGF that crosses the glomerular basement membrane and maintains the fenestrated glomerular endothelium. Together, these structures form the glomerular filtration barrier. A subtle balance of local VEGF production is required for normal glomerular function. Overexpression of podocyte-derived VEGF in the glomerulus causes a collapsing glomerulopathy (28), while inhibition of podocyte VEGF disrupts the filtration barrier, causing protein leak and glomerular thrombotic microangiopathy (TMA) (29).

Glomerular TMA is also the pathological hallmark of hemolytic uremic syndrome (HUS) (25), the clinical triad of acute kidney injury, microangiopathic hemolytic anemia, and thrombocytopenia. A proportion of familial atypical HUS (aHUS) cases result from complement protein mutations, particularly CFH (25), which leads to overactivation of complement in the glomerulus, causing TMA.

Since both the outer retina and renal glomerulus synthesize local VEGF, are sensitive to changes in VEGF, and are vulnerable to genetic variation in CFH and complement activation, we hypothesized that VEGF could regulate local complement activity by inducing CFH synthesis. Here, we show that reduction in VEGF through pharmacological inhibition or genetic ablation decreases local CFH and other complement regulators in both the retina and renal glomerulus, potentially making these sites vulnerable to complement activation.

## Results

### Genetic VEGF ablation inhibits local CFH synthesis in the kidney and eye.

**Figure 1:**
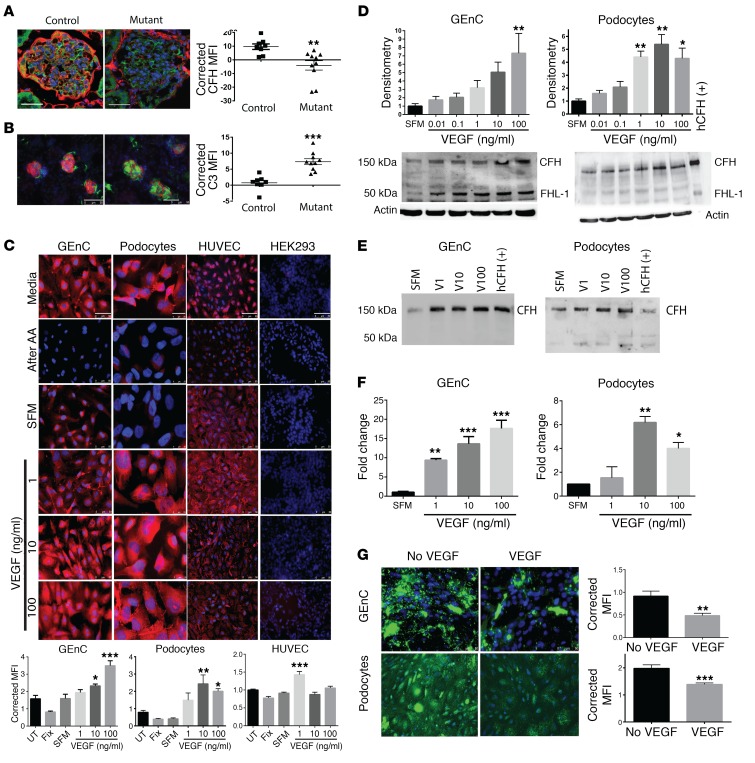
VEGF regulates CFH expression in the glomerulus. Adult mice with an induced deletion of podocyte *Vegfa* showed reduced CFH staining in their glomeruli compared with control mice (**A**, CFH red, podocin green, DAPI blue) and showed glomerular C3 staining (**B**, C3 green, podocin red, DAPI blue). Human GEnC and podocytes stained positively for CFH (red, DAPI blue) under normal cell culture conditions (**C**, media). This was removed by 0.1 M acetic acid treatment (**C**, after AA). CFH staining recurred after 24 hours in serum-free media (SFM), and this was significantly increased by VEGF treatment in a dose-dependent manner. HUVECs also showed CFH staining, but they showed a different response to VEGF treatment (**C**). HEK293 cells did not show CFH staining, and there was no change with VEGF treatment (**C**). Immunofluorescent studies were validated using Western blotting of cell lysates (**D**, *n* = 4), condition media (**E**, representative, *n* = 4), and qPCR (**F**, *n* = 4) shown for GEnC and podocytes (**D** and **F**, *n* = 4, 1-way ANOVA). Twenty-four hours of VEGF treatment also reduced GEnC and podocyte C3d (**G**) (green, DAPI blue) deposits after cell-surface complement activation. (**A** and **B**) *n* = 8–10/group. 20 glomeruli/animal imaged for each antibody tested and averaged. Unpaired, 2-tailed *t* test. (**C** and **G**) Representative images shown from 4 independent experiments. Ten images obtained for each condition. MFI was calculated for CFH/C3d and corrected for cell number determined by DAPI-stained nuclei to semi-quantitatively compare expression. One-way ANOVA with Bonferroni’s post hoc analysis. Scale bars: 10 μm (**A**); 50 μm (**B**, GEnC); 25 μm (**B**, podocytes); 50 μm (**B**, HUVEC); 50 μm (**B**, HEK293); 50 μm (**C**); 50 μm (**G**). *P < 0.05; **P < 0.01; ***P < 0.001.

To study the effects of reduced VEGF levels on complement, glomerular VEGF was ablated in adult mice using a podocyte-inducible Cre as previously reported (29). Fourteen days after VEGF knockdown, mice developed TMA (29), reduced glomerular CFH staining (Figure 1A, controls shown in Supplemental Figure 1, A–C; supplemental material available online with this article; doi:10.1172/JCI86418DS1), and glomerular deposits of C3 that were suggestive of complement activity (Figure 1B). To further examine these results, human glomerular endothelial cells and podocytes were studied and were shown to synthesize inhibitory CFH in response to VEGF in a dose-dependent manner (Figure 1C). The production of CFH was confirmed by Western blotting (Figure 1D, cell lysate; Figure 1E, condition media) and by quantitative PCR (qPCR) using CFH-specific primers (Figure 1F). VEGF did not have this effect on CFH in other endothelial and control cell lines, suggesting the observations were specific to the glomerular cells (Figure 1C, HUVEC and HEK293 panels). Furthermore, 24 hours of VEGF pretreatment reduced complement deposition on these cells after in vitro complement activation (Figure 1G). This suggests that the VEGF-induced changes were functionally relevant.

CFH is considered the most important inhibitor of the alternative pathway (30). To determine why VEGF also reduced C4/C4d deposition (Supplemental Figure 1, D and E), we studied other complement regulators. In the glomeruli of mice with inducible, podocyte-specific *Vegfa* deletion, CD59a was also reduced, but CD55 and Crry were not (Supplemental Figure 1, F–H). CD46 was not examined in mice, since it is only expressed in the testes (31). Human glomerular endothelial cells expressed more CD59 and CD46, but not CD55, after VEGF treatment (Supplemental Figure 1, I–K). These effects were dose dependent.

**Figure 2:**
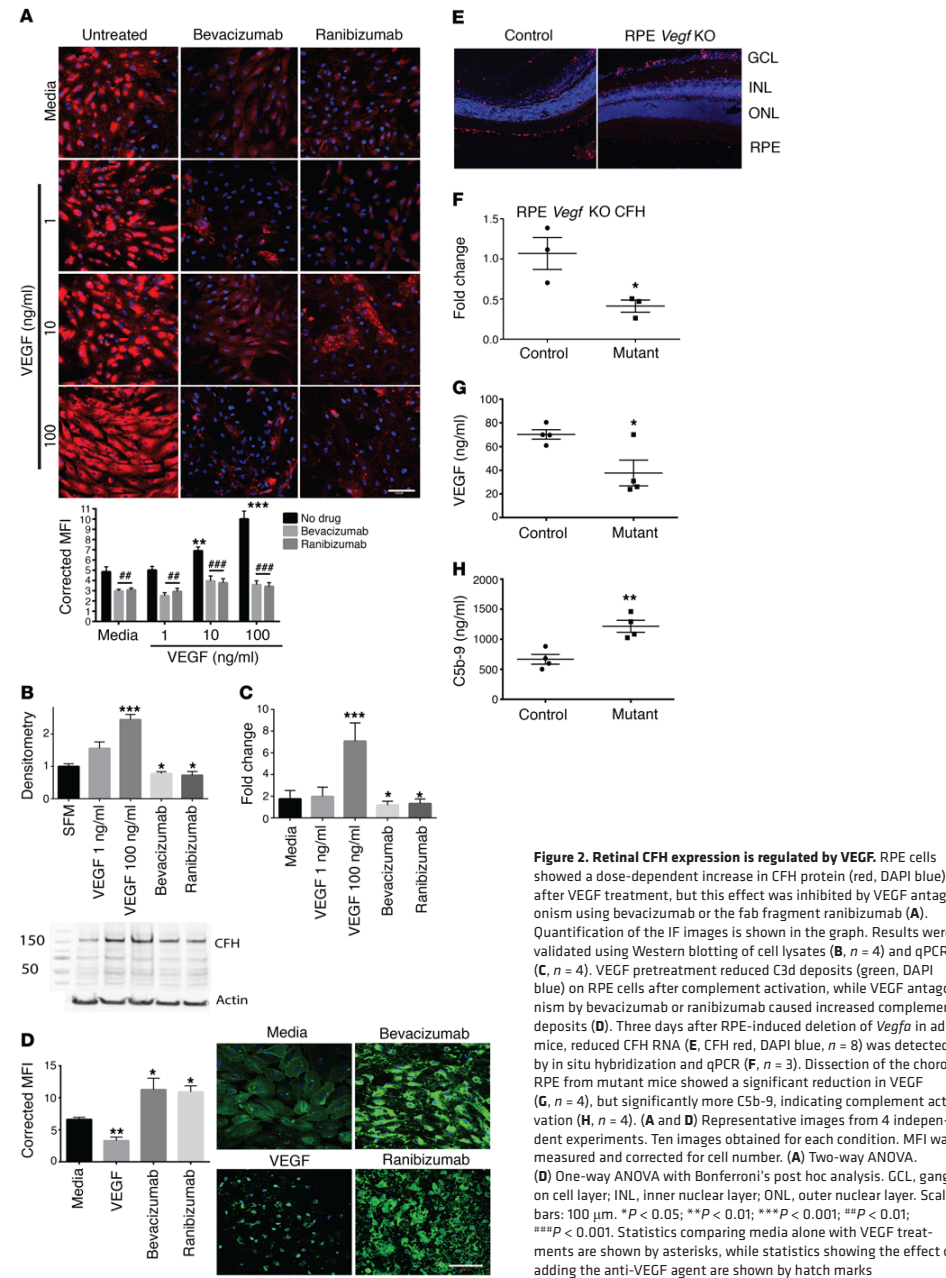
Retinal CFH expression is regulated by VEGF. RPE cells showed a dose-dependent increase in CFH protein (red, DAPI blue) after VEGF treatment, but this effect was inhibited by VEGF antagonism using bevacizumab or the fab fragment ranibizumab (**A**). Quantification of the IF images is shown in the graph. Results were validated using Western blotting of cell lysates (**B**, *n* = 4) and qPCR (**C**, *n* = 4). VEGF pretreatment reduced C3d deposits (green, DAPI blue) on RPE cells after complement activation, while VEGF antagonism by bevacizumab or ranibizumab caused increased complement deposits (**D**). Three days after RPE-induced deletion of *Vegfa* in adult mice, reduced CFH RNA (**E**, CFH red, DAPI blue, *n* = 8) was detected by in situ hybridization and qPCR (**F**, *n* = 3). Dissection of the choroid/RPE from mutant mice showed a significant reduction in VEGF (**G**, *n* = 4), but significantly more C5b-9, indicating complement activation (**H**, *n* = 4). (**A** and **D**) Representative images from 4 independent experiments. Ten images obtained for each condition. MFI was measured and corrected for cell number. (**A**) Two-way ANOVA. (**D**) One-way ANOVA with Bonferroni’s post hoc analysis. GCL, ganglion cell layer; INL, inner nuclear layer; ONL, outer nuclear layer. Scale bars: 100 μm. *P < 0.05; **P < 0.01; ***P < 0.001; ^##^*P* < 0.01; ^###^*P* < 0.001. Statistics comparing media alone with VEGF treatments are shown by asterisks, while statistics showing the effect of adding the anti-VEGF agent are shown by hatch marks

Similarly, in primary human RPE cells, VEGF caused a dose-dependent increase of CFH and CD46, but not CD55 (Figure 2, A–C and Supplemental Figure 2, A and B), with reduced complement deposits (Figure 2D and Supplemental Figure 2C). The same results were obtained from ARPE19 cells, but only primary human RPE cell data are shown here. VEGF antagonism using bevacizumab or the fab fragment ranibizumab reduced CFH and CD46 expression (Figures 2, A–C and Supplemental Figure 2, A and B) and increased C3d and C4d deposits (Figure 2D and Supplemental Figure 2C). The doses of VEGF antagonist used were comparable to those given to patients by intravitreal injection. To determine whether these effects also occurred in vivo, RPE-induced *Vegfa* knockout was accomplished in adult mice using VMD2-Cre. These mice showed severe choriocapillaris attenuation (4) and, similarly to mice with inducible, podocyte-specific *Vegfa*, complement inhibitors CFH and CD59a were reduced 3 days after induction (Figure 2, E and F, and Supplemental Figure 2D). Reduction in VEGF was confirmed in choroid/RPE sections from RPE-induced *Vegfa*-knockout mice (Figure 2G). These same sections showed increased C5b-9 levels compared with controls, showing that they had increased complement activation (Figure 2H). Conversely, 3 days after RPE-induced *Vhl* knockout, which causes pseudo-hypoxia and increased RPE-derived VEGF (32), more CFH was detected (Supplemental Figure 2E). These effects were not limited to the outer retina. Other sources of retinal VEGF are amacrine and horizontal cells, lateral interneurons that associate with intraretinal capillaries (10). When *Vegfa* and *Vhl* were knocked out of these neurons, similar changes in CFH were detected in the inner retina, showing an in vivo dose response (Supplemental Figure 2, F and G). Reductions in CD55 and CD59a, but not Crry, were also noted in these mice (Supplemental Figure 2H). These results suggest that VEGF regulation of CFH and other complement inhibitors is a common mechanism in the retina and thus may have implications for the pathogenesis of other retinal diseases, e.g., diabetic retinopathy. Furthermore, we showed that VEGF reduction in the glomerulus or the retina was associated with reduced CFH, complement deposition, and vascular changes, suggesting that local complement regulation is important for both these sites.

### VEGF-induced CFH changes are mediated by PKC signaling.

**Figure 3:**
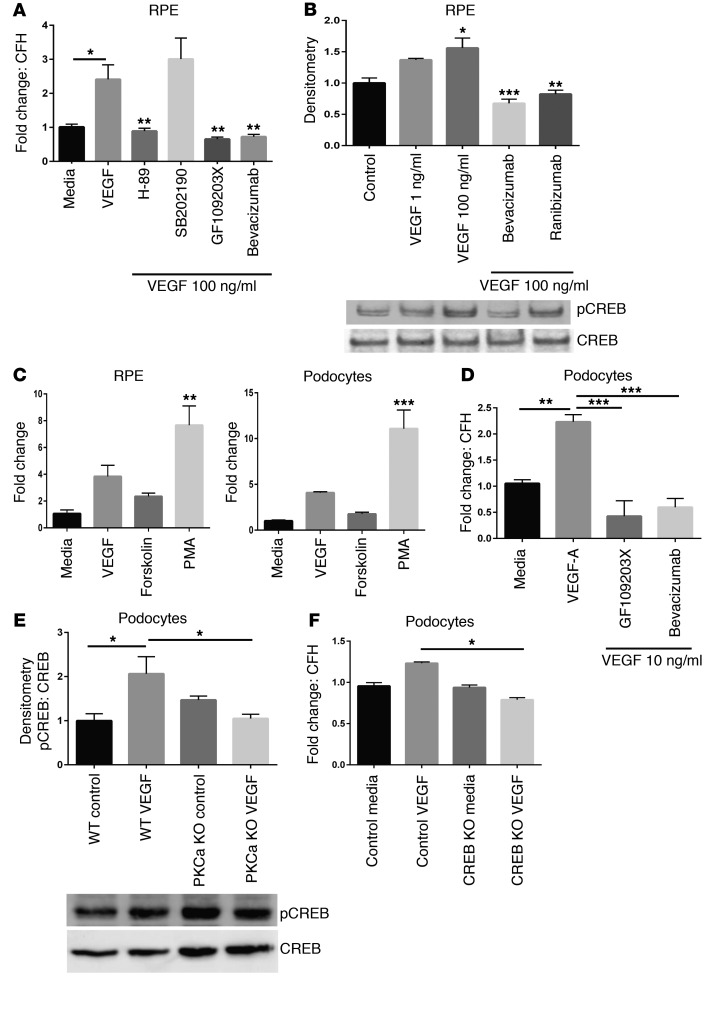
VEGF-induced changes in CFH were mediated by PKC signaling via CREB. RPE cells treated with either PKC (GF109203X) or PKA inhibitor (H-89) before treatment with VEGF failed to produce an increase in CFH RNA transcript when compared with VEGF treatment alone. This was comparable to the effect of bevacizumab and VEGF treatment (**A**, *n* = 4). Inhibiting p38 MAPK (SB202190) prior to VEGF stimulation had no effect on the increase in CFH transcript. VEGF caused greater phosphorylation of the CREB transcription factor (top panel), while anti-VEGF treatment reduced this phosphorylation (**B**, *n* = 4). Total CREB was also measured in these samples (lower panel). Human RPE cells and podocytes treated with the PKC activator (PMA) or PKA/adenylate cyclase activator (forskolin) showed increased CFH transcript compared with control (**C**, *n* = 3). The effect was more pronounced with PKC activation. Human podocytes were then treated with PKC inhibitor (GF109203X) or bevacizumab prior to VEGF stimulation, and they also showed reduced CFH RNA compared with VEGF treatment alone (**D**, *n* = 3). Podocytes with a CRISPR-induced knockout of PKC-α showed reduced CREB phosphorylation after VEGF stimulation compared with control cells (**E**, *n* = 3), showing that podocyte VEGF stimulation produced CREB phosphorylation via PKC-α. Finally, podocytes with a CRISPR-induced knockout of CREB showed significantly reduced CFH RNA compared with control VEGF–stimulated podocytes (**F**, *n* = 3). One-way ANOVA. *P < 0.05; **P < 0.01; ***P < 0.001.

To begin to examine the molecular mechanisms underlying the CFH expression changes, human RPE cells were treated with several inhibitors of pathways downstream of VEGF/VEGFR2 signaling (33, 34) prior to VEGF stimulation. Pharmacological inhibition of PKC (GF109203X) and PKA (H-89), but not p38 MAPK (SB202190), prevented the VEGF-induced increase in CFH transcript (Figure 3A). The reduction was comparable to that produced by bevacizumab. Both PKA and PKC phosphorylate the transcription factor CREB (35), which previous studies showed has putative responsive elements in the human CFH promoter (36). CREB phosphorylation was shown to be sensitive to VEGF supplementation or inhibition (Figure 3B). Conversely, PKC activation (phorbol 12-myristate 13-acetate [PMA]) and, to a lesser extent, PKA activation (forskolin), increased CFH transcripts in both podocytes and RPE cells (Figure 3C). Podocytes were also tested with the PKC inhibitor (GF109203X) and bevacizumab prior to VEGF stimulation, and a reduction in CFH RNA was detected (Figure 3D). To further examine this mechanism, and because the greatest effects were seen with PKC modulation, a CRISPR/Cas9 PKC-α knockout podocyte cell line was made. In contrast to control podocytes, PKC-α knockout reduced CREB phosphorylation after VEGF stimulation (Figure 3E), further suggesting that VEGF-induced CREB phosphorylation was mediated by PKC-α. Next, we tested podocytes with a CRISPR/Cas9-induced CREB knockout and found that, compared with control podocytes, these cells produced significantly less CFH RNA after VEGF stimulation (Figure 3F). Together, these data suggest that the VEGF-induced increase in CFH is mediated via PKC-α/CREB signaling. Further work is needed to confirm these results and probe further into the mechanisms responsible for the differential response observed between endothelial cells.

### Intravitreal anti-VEGF injection induced complement activation in the retina.

Having shown that genetic VEGF manipulation altered CFH expression, we sought to determine whether pharmacological VEGF reduction could phenocopy the results. Since bevacizumab and ranibizumab target human VEGF, we used a monoclonal antibody against murine VEGF that was validated prior to use (Supplemental Figure 3, A–C).

**Figure 4:**
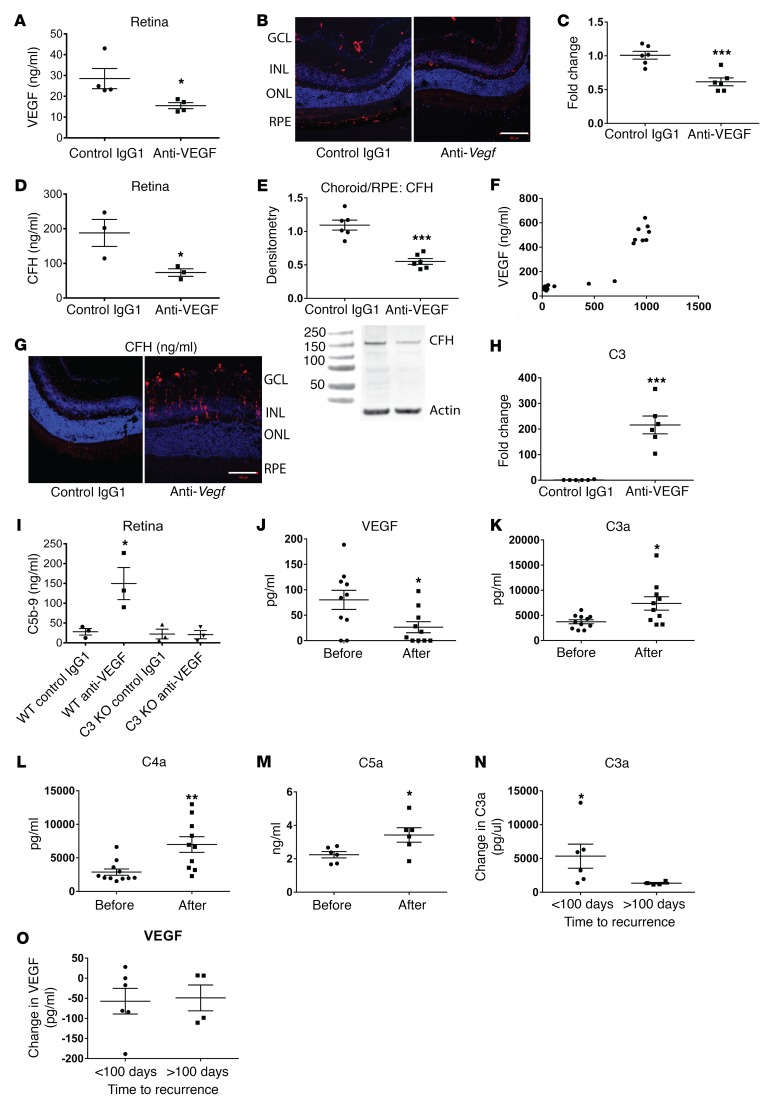
Intravitreal anti-VEGF results in reduced CFH and complement activation. The retinas of mice injected with anti-mouse VEGF showed a significant reduction in VEGF protein after 48 hours (**A**, *n* = 4). This was associated with a reduction in CFH RNA, as shown by in situ hybridization (**B**, CFH red, DAPI blue, *n* = 8) and confirmed by qPCR (**C**, *n* = 6), CFH protein in the retina (**D**, *n* = 3), and choroid/RPE (**E**, 150 kDa band, *n* = 6). VEGF and CFH concentrations measured in the same lysates showed significant correlation with a Pearson coefficient of 0.94 (95th CI 0.87–0.97) (**F**, *n* = 30). There was increased C3 RNA after a single anti-VEGF injection (**G**, *n* = 8). A linear C3 staining pattern in the in situ hybridization suggests that Müller cells may be a C3 source (C3 red, DAPI blue). A 200-fold increase in retinal C3 RNA was confirmed by qPCR (**H**, *n* = 6). WT mice injected with anti-VEGF also showed increased C5b-9, indicating complement activation (**I**, *n* = 3). C3 knockout mice did not show this effect. The changes in RNA were detected after 24 hours, while protein changes were detected after 48 hours. Aqueous humor from 10 ARMD patient eyes was sampled before and 48 hours after a single intravitreal bevacizumab. The samples obtained after injection showed reduced VEGF (**J**) and increased C3a (**K**), C4a (**L**), and C5a levels (**M**). Patients who had a recurrence of wet ARMD within 100 days of treatment (range 47–86 days, *n* = 6) had a greater rise in C3a 48 hours after intravitreal bevacizumab injection than patients who relapsed more than 100 days later (range 152–449days, *n* = 4) (**N**). There were no significant differences in VEGF (**O**). Human samples were analyzed by CBA or ELISA carried out in triplicate and analyzed using Mann-Whitney *U* test. Images represent 3 independent experiments. Scale bars: 100 μm. Unpaired, 2-tailed *t* test (**A**, **C**, **D**, **E**, **H**, **J**–**O**); 1-way ANOVA (**I**). *P < 0.05; **P < 0.01; ***P < 0.001.

After administering a single dose of anti-VEGF intravitreally to adult mice, we confirmed reduction in retinal VEGF concentrations (Figure 4A) and observed reduced retinal CFH 24 to 48 hours later (Figure 4B, in situ hybridization; control in Supplemental Figure 4A, C-qPCR, D-CFH ELISA) and reduced CFH in choroid/RPE samples (Figure 4E, Western blot). We also used Western blotting to study retinal lysates and detected several bands that could represent CFH-related proteins (Supplemental Figure 4C). These bands were not detected in choroid/RPE lysates. Reduced retinal CFH was also seen after injection of commercially available aflibercept (Supplemental Figure 4D), but not after injection with PBS or control IgG1 (Supplemental Figure 4, A and D). Interestingly, VEGF and CFH levels were also reduced in the kidneys of mice injected with intravitreal anti-VEGF 48 hours later (Supplemental Figure 5, A and B). VEGF and CFH concentrations measured in the same samples were correlated and showed a Pearson correlation coefficient of 0.94, indicating a strong correlation (Figure 4F). Retinal C3 was also increased 24 hours after injection (Figure 4G, in situ hybridization; control in Supplemental Figure 4B, H-qPCR). To directly assess complement activation in these mice, we measured C5b-9. This was significantly increased 48 hours after anti-VEGF injection in WT mice, but not in C3-knockout mice (Figure 4I). To validate these murine studies, aqueous humor from ARMD patients naive to anti-VEGF therapy was analyzed before and 48 hours after a single injection of intravitreal bevacizumab. First, a significant reduction in VEGF was confirmed (Figure 4J). Next, complement activation was demonstrated with elevated C3a, C4a, and C5a levels (Figure 4, K–M). In this small patient group, a greater increase in C3a levels 48 hours after 1 intravitreal injection of bevacizumab correlated with an earlier time to recurrence of wet ARMD (Figure 4N). No differences in VEGF reduction (Figure 4O) or in C4a or C5a levels were detected between these groups (Supplemental Figure 6, A–C).

After detecting the anaphylatoxins C3a, C4a, and C5a, which stimulate inflammation, we also measured proinflammatory cytokines. IL-6 and IL-8 were also increased in these patient samples and primary human RPE cell culture supernatant and in murine retinae after intravitreal anti-mouse VEGF, but not control IgG1 (Supplemental Figure 7, A–C). This was noteworthy because increased IL-6 and IL-8 have been associated with wet ARMD (37, 38). Retinal inflammation was further demonstrated by Müller glia and microglia activation, while apoptotic cells were detected in the ganglion cell layer after a single intravitreal dose of anti-VEGF (Supplemental Figure 7, D–F). Aflibercept intravitreal injection produced similar effects. This demonstrates that a single dose of VEGF antagonist can induce complement activation and inflammation.

### Complement pathway inhibition reduced anti-VEGF–induced endothelial cell activation.

**Figure 5:**
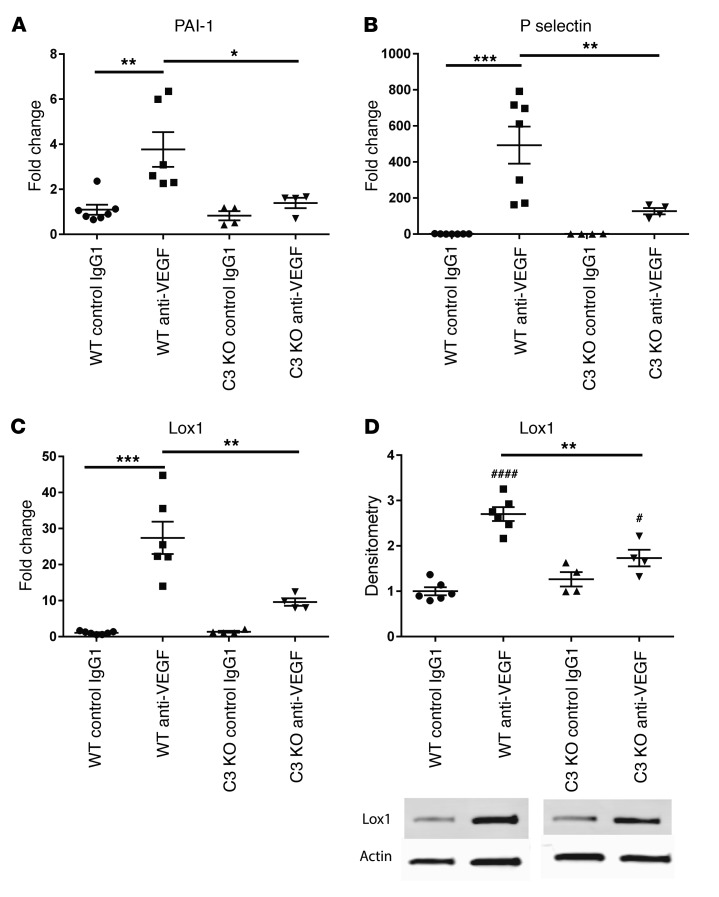
Intravitreal anti-VEGF causes endothelial activation, but this is reduced by complement inhibition. Evidence of endothelial cell activation was detected 24 hours after a single dose of intravitreal anti-mouse VEGF, as shown by an increase in PAI-1/serpine 1 (**A**), P-selectin (**B**), and oxidized low density lipoprotein receptor 1 (Lox1) (**C**) in WT mice. Complement C3 knockout mice showed significantly less PAI-1, P-selectin, and Lox1 after intravitreal anti-VEGF when compared with WT mice (**A**, **B**, **C**, comparison shown by black bar). Lox 1 protein was also increased on Western blot 48 hours after single intravitreal injection of anti-VEGF (**D**, first 2 columns), but this increase was significantly less in C3 knockout mice at the same time point when compared with WT mice (**D**, WT anti-VEGF vs. C3KO anti-VEGF). This suggests that complement inhibition may reduce levels of endothelial cell activation after anti-VEGF injection. *n* = 4–6 mice per condition. qPCR run in triplicate. One-way ANOVA. *P < 0.05; **P < 0.01; ***P < 0.001; ^#^*P* < 0.05; ^####^*P* < 0.0001. Asterisks show statistics comparing the anti VEGF effect between wild type and C3 KO mice. The hatch marks show statistics comparing wild type mice only (IgG1 control vs anti VEGF) or C3 ko mice only (IgG1 control vs anti VEGF).

Since genetic ablation of RPE VEGF caused degeneration of the choriocapillaris, we studied the effect of intravitreal anti-VEGF on markers of endothelial cell activation. Murine eyes showed increased levels of serpine-1/endothelial plasminogen activator inhibitor (PAI-1), P-selectin, and lectin-like oxidized LDL receptor (39) 24 to 48 hours after 1 anti-VEGF injection (Figure 5, A–D). To determine whether complement activation contributed to this, mice lacking the complement-activating protein C3 were studied. These mice showed significantly reduced markers of endothelial activation after intravitreal anti-VEGF compared with controls (Figure 5, A–D). This suggests that complement inhibition reduced endothelial cell activation that resulted from anti-VEGF injection. C3-knockout mice showed no difference in apoptosis, Müller glia, or microglia cell activation after VEGF antagonism (Supplemental Figure 7, D–F). Therefore, complement activation may be particularly important for anti-VEGF–associated endothelial cell changes.

### CFH genetic variants caused more complement deposits, which were increased by VEGF antagonism.

**Figure 6:**
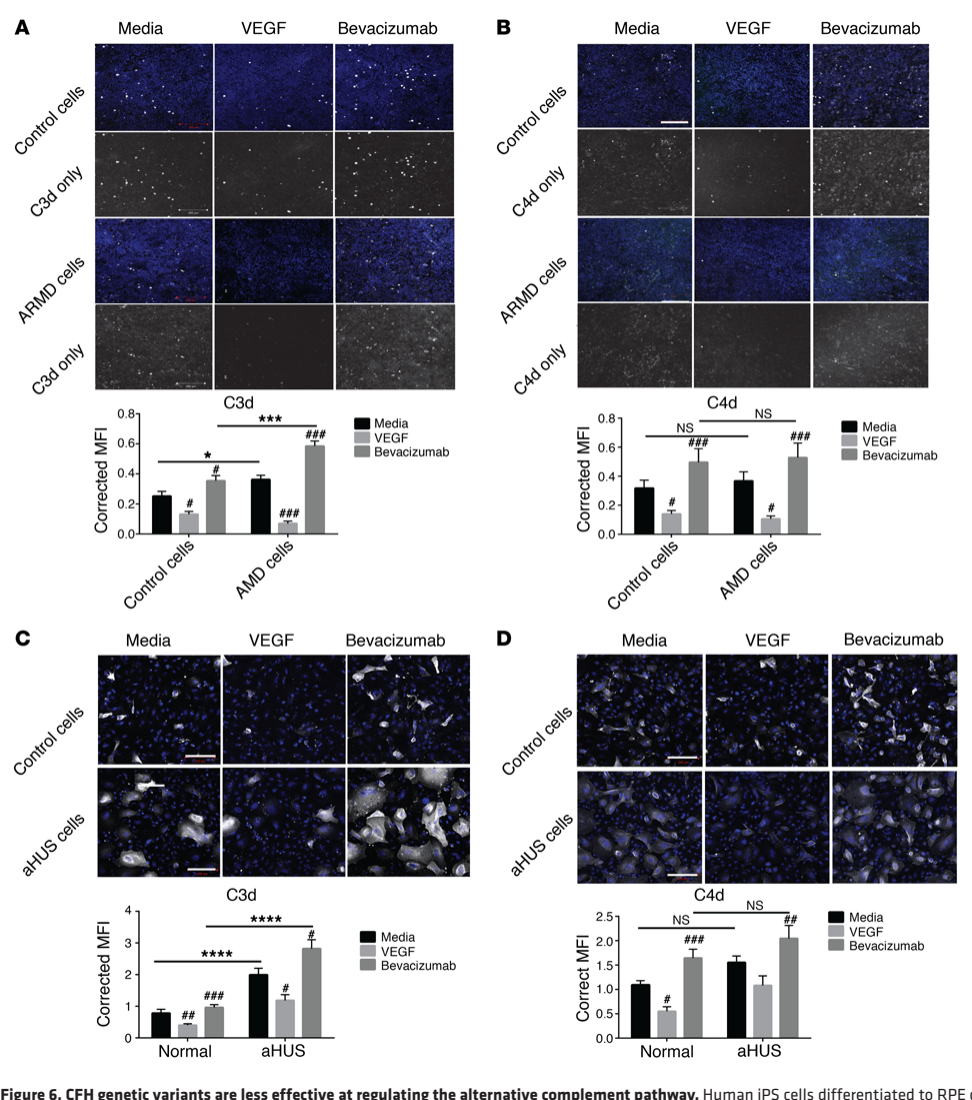
CFH genetic variants are less effective at regulating the alternative complement pathway. Human iPS cells differentiated to RPE containing the ARMD CFH 402H polymorphism (ARMD cells) showed greater C3d deposits (C3d white, DAPI blue) compared with control cells when complement was activated on the cell surface (**A**, media, untreated column comparison). This effect was exacerbated by bevacizumab treatment (**A**, bevacizumab column comparison). There was no difference between cell types when C4d staining was analyzed at baseline without treatment or after bevacizumab (**B**, black lines denote comparison, C4d white, DAPI blue). However, bevacizumab significantly increased C4d deposits, and VEGF reduced these on both cell lines. The same effect was seen in podocytes after treatment with VEGF and bevacizumab for C3d (**C**, C3d white, DAPI blue) and C4d (**D**, C4d white, DAPI blue). Representative images from 4 independent experiments. Ten images obtained for each condition per individual experiment. Fluorescence was measured as MFI and corrected for cell number (semi-quantitative assessment). Two-way ANOVA. Statistics comparing the same cell types exposed to different treatments denoted as hatch marks. Statistics comparing different cell types denoted as NS or asterisks. ^#^*P* < 0.05; ^##^*P* < 0.01; ^###^*P* < 0.001; *P < 0.05; ***P < 0.001; ****P < 0.0001. Black bars show which conditions were compared. Scale bars: 200 μm

To study the effects of VEGF antagonism in patients with CFH genetic variants and how these may alter complement cell-surface regulation, we studied RPE that carried the ARMD-associated CFH 402H polymorphism (ARMD RPE) and podocytes from an aHUS patient carrying a known CFH mutation (Arg1182Ser) (aHUS podocytes) (40). First, we measured CFH production by these cells at baseline and after treatment with VEGF, bevacizumab, or aflibercept and correlated this with VEGF concentration in serum-free conditioned media (Supplemental Figure 8, A and B). A strong correlation between CFH and VEGF was found in both induced pluripotent stem cells (iPS cells) RPE (Supplemental Figure 8A, *r* = 0.86) and podocytes (Supplemental Figure 8B, *r* = 0.87). Next, functional complement studies were performed. Without treatment, ARMD RPE showed significantly more C3d deposits compared with controls (Figure 6A), but there were no significant differences in C4d deposits (Figure 6B). This suggests increased activation of the alternative pathway, but no difference in classical pathway regulation on the surface of these ARMD cells that possess the CFH polymorphism. Bevacizumab significantly increased C3d and C4d deposits in both cell lines, while VEGF treatment reduced both (Figure 6, A and B, hatch marks). This confirmed our previous results using primary human RPE cells (Supplemental Figure 2). However, there was significantly (Figure 6A, asterisks) more C3d on the ARMD cells after bevacizumab compared with untreated ARMD cells, suggesting that the effects of VEGF antagonism on complement inhibitor expression further reduced the complement regulatory capacity of the ARMD cells. Treatment with aflibercept showed comparable results (Supplemental Figure 8, C and D). Similar results were also obtained in human aHUS podocytes expressing Arg1182Ser CFH mutation that affects CFH cell-surface binding (40) (Figure 6, C and D and Supplemental Figure 8E). This suggests that ARMD RPE and aHUS podocytes are less efficient at inhibiting the alternative pathway at the cell surface and so could be more susceptible to reduction of CFH and other complement inhibitors mediated by VEGF antagonism. This may explain why patients with CFH genetic variants are at greater risk of ARMD and HUS and could have implications for patients receiving anti-VEGF therapy.

## Discussion

**Figure 7:**
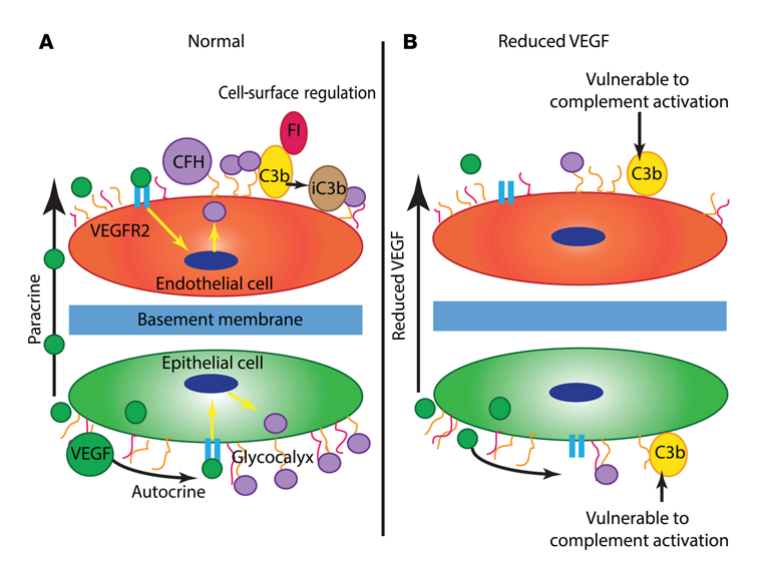
Proposed model of how VEGF regulates local complement activity. (**A**) Under normal circumstances in the outer retina and renal glomerulus, specialized epithelial cells (the RPE and podocytes, respectively) produce VEGF that has both autocrine effects and paracrine effects on neighboring endothelial cells. VEGF signaling through VEGFR2 causes inhibitory complement proteins such as CFH to be produced by these cells. These complement inhibitors function at the cell surface to prevent complement activation caused by spontaneous alternative pathway hydrolysis. In the case of CFH, this occurs by binding to the cellular glycocalyx and acting as a cofactor for factor I (FI). (**B**) When anti-VEGF therapy is given, there is reduced local VEGF production, which leads to less VEGFR2 signaling. This may cause a reduction in local complement inhibitor synthesis and secretion, making the cells more vulnerable to complement activation.

For the first time, to our knowledge, we report a link between local VEGF availability and complement inhibition in the retina and renal glomerulus that is mediated by changes in local complement inhibitor expression (Figure 7). This provides insight into the underlying pathogenesis of ARMD and HUS as well as providing a possible explanation for the adverse effects associated with VEGF antagonism in both organs.

ARMD is a multifactorial disease. Aging is the primary risk factor, but genetic susceptibility and environmental exposures influence disease acquisition (1). Complement gene mutations confer risk (1), but it is unclear precisely how they contribute to ARMD pathogenesis. The most widely studied is the CFH 402H polymorphism, and we demonstrated that RPE cells with this polymorphism showed more cell-surface complement deposition. This supports previous studies that showed CFH 402H does not effectively bind to the cell-surface glycosaminoglycan heparan sulphate and this can affect surface complement regulation (24, 41, 42). In the kidney, several aHUS CFH mutations also affect the protein’s ability to bind to the cell surface and regulate complement (43). Therefore, we compared an aHUS podocyte cell line with a known CFH mutation (40) to the ARMD RPE cells with the CFH polymorphism in our complement activation assay and obtained similar results, suggesting that the effect observed in RPE cells could be due to impaired cell-surface binding of CFH 402H. Formal binding studies would be needed to confirm these results.

Complement activation is a known feature of ARMD (21, 23, 24, 44), and our experiments suggest VEGF antagonism could exacerbate this by reducing synthesis of CFH and other inhibitory complement proteins. Since both C3 and C4 deposits were increased by VEGF antagonism, these effects likely represent changes in multiple complement inhibitors simultaneously. These effects were more pronounced in cells expressing CFH 402H, possibly because they already have reduced complement regulatory capacity and anti-VEGF treatment could decrease this further. This could explain why the CFH 402H polymorphism has been reported to correlate with a reduced response to anti-VEGF therapy (19, 45, 46), although in this complex disease, it is likely that several other factors also contribute to the variable response to anti-VEGF therapy (47). Furthermore, while controversial, there are patient studies suggesting that VEGF antagonists may enhance progression of GA (8, 9, 48, 49), which could be related to direct complement-mediated damage of the RPE cells. Importantly, this does not affect every patient and more work is needed to identify those at risk, but homozygosity of the CFH 402H polymorphism may be one factor.

Complement activation may also affect the choroidal vasculature (50). Our studies showed that inhibiting complement partially prevented the anti-VEGF–induced increase in endothelial cell activation, suggesting that complement inhibition could protect the endothelium. Previously, animal models showed that alternative pathway activation contributes to the development of CNV (51, 52), while cell culture studies revealed that sublytic levels of MAC can increase RPE-derived VEGF (44), which could contribute to the development of CNV. Considering these data, early complement inhibition in ARMD may prevent some of these negative effects and could reduce the abnormal increase in secreted RPE-derived VEGF. However, a balance must be struck, since murine studies also suggest that complete, prolonged complement inhibition is detrimental (53). Further work is needed to examine this, particularly in humans, since there are important species differences in the complement cascade. Complement inhibition in ARMD, including GA, is an area of active study, with several agents currently in phase 2/3 trials (54).

Complement protein mutations are also associated with glomerular TMA, as shown by studies of familial aHUS (55). The same glomerular pathology was later identified in patients receiving systemic bevacizumab to treat tumor angiogenesis (56, 57). Eremina et al. showed that these effects were replicated in mice with a glomerular-specific VEGF knockout (29), highlighting the importance of local podocyte-derived VEGF in the maintenance of the glomerular endothelium. Using the same model and human cells in vitro, we show that reduced glomerular VEGF decreased expression of local CFH and other complement regulators in both podocytes and glomerular endothelial cells, predisposing them to complement deposition. These findings link the pathogenesis of glomerular TMA associated with anti-VEGF therapy to that of complement-mediated aHUS and may explain why the renal glomerulus is susceptible to complement-mediated disease. Interestingly, preeclampsia, another glomerular disease associated with endotheliosis and caused by chronic VEGF inhibition secondary to increased soluble VEGFR1, is also associated with complement activation (58, 59), suggesting that this work may also be relevant to this disease.

Glomerular TMA also occurs after administration of receptor tyrosine kinase inhibitors such as sunitinib that block VEGFR2 (57, 60) and in mice with reduced endothelial cell VEGFR2 expression (61), suggesting that anti-VEGF–associated TMA results from decreased VEGFR2 signaling. Building on this, we show that inhibiting PKC blocked the VEGF-induced CFH increase. This was confirmed with CRISPR knockout studies targeting PKC-α and CREB. This is not the first time these pathways have been implicated in complement protein regulation. While CFH was not studied, Mason et al. showed that HUVECs and dermal endothelial cells expressed more CD55, but not CD46 or CD59, after VEGF treatment (62). This was mediated by PKC-CREB signaling (63). In our study, HUVECs exhibited a different response to VEGF-induced CFH expression compared with glomerular endothelial cells. Conversely, the glomerular endothelial cells did not show an increase in CD55 after VEGF treatment, but CD46 and CD59 were both increased along with CFH. This could indicate a heterogeneous endothelial response to VEGF and complement protein expression that varies between different vascular beds. While further work is needed to examine this in more detail, previous studies showed different isoforms of PKC can produce differential effects in endothelial cells (64). However, VEGF/VEGFR2 signaling is complex (34) and there are several other points at which modulation could occur: (a) interactions between receptor and coreceptors such as the neuropilins (34, 65) or extracellular matrix molecules such as integrins (66) or the cellular glycocalyx (67); (b) receptor internalization, recycling, and degradation may also be important (65, 68, 69); and (c) differential phosphorylation could occur secondary to the availability of adapter and scaffolding proteins as well as phosphatases such as PTP1B (65, 69). Furthermore, there are several ways CREB phosphorylation can be modified (70). These processes could explain the differential effects of VEGF at different sites around the body, but an extensive systematic study would be needed to tease out the details.

VEGF antagonists are widely used to treat not only ARMD but also cancers and other diseases associated with retinal neovascularization, including proliferative diabetic retinopathy, retinal vein occlusion (71), and, “off label,” for retinopathy of prematurity (72). Intravitreal doses are smaller than systemic ones and thus would be expected to cause fewer adverse effects. However, the drugs are systemically cleared and can suppress circulating VEGF levels (73). We found reduced VEGF and CFH levels in murine kidneys 48 hours after intravitreal injection. This confirmed primate studies that detected anti-VEGF agents in glomeruli one day after intravitreal injection (74). Furthermore, glomerular dysfunction has been reported for all intravitreal VEGF antagonists currently used in patients (75, 76). Not all patients are affected, but preexisting renal pathology may increase susceptibility. Recently, 2 renal transplant patients were reported to have developed antibody-mediated rejection after intravitreal therapy for ARMD (75). Another study reported diabetic patients with nephropathy and retinopathy whose renal function deteriorated from stage IV to stage V chronic kidney disease after intravitreal anti-VEGF (76). Therefore, documentation of renal function, blood pressure, and urinalysis before and during treatment with anti-VEGF agents would be prudent, particularly in ARMD patients who have renal comorbidities. From our results, patients carrying CFH genetic variants may also be more sensitive to VEGF antagonism. The ARMD CFH 402H polymorphism has been associated with membranoproliferative glomerulonephritis (43), which could indicate that the kidneys of these patients are more vulnerable to complement activation as well. To confirm these findings, a larger study detailing genotype/phenotype correlations of renal and retinal function before and after VEGF antagonism would be beneficial to identify factors that predispose patients to developing these adverse effects. This could potentially guide an individualized approach to therapy in the future. In the meantime, our findings suggest that side-effect monitoring should be more rigorous. Assessment of glomerular function by monitoring for proteinuria, hypertension, and reduced renal function could be particularly important for patients with preexisting renal disease or a CFH genetic variant receiving anti-VEGF therapy. In ARMD, documentation of visual acuity and GA before and, at regular intervals, after initiation of VEGF antagonism may help guide therapy, including determining whether it should be continued or stopped. With increasing use of VEGF antagonists, all clinicians should be aware of the potential adverse effects and be vigilant to aid detection and prevent unnecessary morbidity.

## Methods

Additional supplemental methods used are detailed in the supplemental materials.

### Human samples.

After written informed consent, 10 ARMD patient eyes naive to anti-VEGF therapy had aqueous humor sampled before intravitreal bevacizumab injection (1.25 mg) and 48 hours later by Yoshihiro Wakabayashi or Yasuyuki Yamauchi (Tokyo Medical University Hospital). Eight patients were studied, 5 male and 3 female, with an average age of 71.1 ± 3.2 years. All had typical ARMD diagnosed after fundus examination with a fluorescein angiography, indocyanine green angiography, and optical coherence tomography. Wet ARMD was diagnosed according to established criteria (77). The eyes studied showed classic CNV (2/10), minimally classic CNV (1/10), and occult CNV (7/10), but no retinal angiomatous proliferation. Patients with cataracts causing moderate-to-severe visual disturbance received elective surgery 2 days after intravitreal bevacizumab. Aqueous humor was collected before surgery for evaluation after injection. Samples were immediately frozen and stored at –80°C until analyzed. Recurrence was defined by fluorescein angiography (recurrence of leakage and leakage from new vessels at the site of previous lesions), indocyanine green angiography (recurrence of leakage), OCT (fluid in OCT and increase of macular thickness in OCT with visual loss), and clinical findings (new macular hemorrhages, new classic CNV) during the follow-up examinations, as previously described (78).

### Mice.

Kidneys from podocyte-specific tetracycline-inducible *Vegfa*-knockout mice (29), aged 3 to 4 weeks (supplied by Susan Quaggin, [Northwestern University, Chicago, Illinois, USA] and Vera Eremina [Samuel Lunenfeld Research Institute, Toronto, Canada]), were obtained 14 days after oral induction using 0.2% doxycycline. During this time, mice were monitored for proteinuria alongside noninduced littermate controls.

Retinal *Vegfa* knockout was achieved in RPE cells using tetracycline inducible VMD2 Cre (4) (Yung-Zheng Le, University of Oklahoma, Oklahoma City, Oklahoma, USA) and in amacrine and horizontal cells using Ptf1a Cre (Jackson Laboratories) (10). Floxed gene ablation was induced in 4-week-old VMD2-Cre *Vegfa* or *Vhl* floxed mice (Jackson Laboratories) by administering 80 μg/g body weight doxycycline intraperitoneally for 3 consecutive days as described (4). Eyes were analyzed 3 days after induction. *Ptf1a* is expressed in the retina from E12.5 (10), and mice were analyzed on postnatal day 18.

The number of mice required for each experiment was determined as described (79) based on a power of 80% and *P* = 0.05. For all studies, equal numbers of males and females were included in each group. No formal randomization was undertaken. Outliers were identified and removed if they were more than 3 SD from the mean.

### Intravitreal murine injections.

Anti-mouse and human VEGF B20 biosimilar was made according to the published sequence of B20-4.1 (80). The variable region of the heavy and light chains of B20-4.1 was fused to the constant region of IgG1 and k chain, respectively. Stable mammalian 293 cell lines for recombinant B20 production were established using the piggyBac transposon system (81). Briefly, the 2 genes encoding for the heavy and light chains were cloned into 2 different PB-T-RfA transposons, and we used a 1:2 ratio of heavy to light chain during transfection. Recombinant B20 was purified using protein A affinity column, and stored in 50 mM sodium phosphate buffer containing 150 mM NaCl (pH 7.0).

Adult C57BL/6 mice or complement C3 knockout mice (Jackson Laboratories) were anesthetized using 15 mg/kg ketamine and 7 mg/kg xylazine administered intraperitoneally prior to 0.5 μl intravitreal injection of 5 mg/ml anti-murine VEGF (B20), control murine IgG1 (R&D Systems), aflibercept (Regeneron Pharmaceuticals), or control human IgG1 (R&D Systems). PBS-injected and uninjected eyes served as further controls. Eyes were extracted at 24 or 48 hours to analyze RNA or protein, respectively. Four to six eyes per condition were analyzed with 3 different litters used per experiment.

### Cell culture.

Human conditionally immortalized glomerular endothelial cells and podocytes transfected with temperature-sensitive SV40 were used as described (Satchell et al., ref. 82; Saleem, et al., ref. 83). Podocyte cells were also obtained from an aHUS patient with an Arg1182Ser (G3546T) CFH mutation, which affects the ability of CFH to bind to heparin and C3b (40). Immortalized cells were grown in humidity with 5% CO_2_ at the permissive temperature of 33°C until 80% confluent, then thermo-switched to 37°C for 4 to 5 days for glomerular endothelial cells (GEnC) and 12 to 14 days for podocytes to “switch off” SV40 expression and facilitate differentiation. Podocytes were grown in RPMI-1640 with 10% FBS supplemented with 1% insulin, transferrin, and selenium. GEnC were grown in EGM-2MV media with 5% FBS supplemented with human EGF (hEGF), hydrocortisone, gentamicin, amphotericin, human FGF-B (hFGF-B), IGF-1, and ascorbic acid (EGM-2MV BulletKit except VEGF, Lonza).

ARPE19 cells (Life Technologies), human primary RPE cells (Lonza), HUVEC cells (Lonza), and HEK293 cells (ATCC) were grown at 37°C in humidity with 5% CO_2_. AREP19 were maintained in DMEM F12 with 2% FBS. HRPE cells were grown in RPE cell basal media (RtEBM) with BulletKit (Lonza). HUVEC cells were grown in EGM2 media with 5% FBS and added BulletKit (Lonza). HEK293 cells were maintained in RPMI 1640 with 5% FBS.

iPS cells differentiated into RPE were supplied by K. Bharti. The human iPS cells were derived and differentiated to RPE as described (84). Cells were genotyped for the CFH Y402H polymorphism (rs1061170, T1277C). Two cell lines were used, one with the normal 402Y allele and one with the ARMD-associated 402H allele. They were maintained in knockout DMEM with 1% glutamate, nonessential amino acids, penicillin and streptomycin, 0.18% β mercaptoethanol, 10 ng/ml basic FGF, and 20% knockout serum replacement (Life Technologies).

### Cell treatments.

Cells grown in serum-containing media were serum starved for 1 to 2 hours prior to treatment with VEGF-165 (0.1 ng/ml-100ng/ml, R&D Systems), bevacizumab (250 μg/ml), ranibizumab (125 μg/ml) (both from Genentech), aflibercept (500 μg/ml, Regeneron), inhibitors, or activators in serum-free media. Wortmannin (0.5 μM) (W1628-PI3Kinase inhibitor), H-89 (20 μM) (B1427-PKA inhibitor), U0126 (10 μM) (U0120-MEK inhibitor), SB202190 (20 μM) (S7067-p38 MAPK inhibitor), GF109203X hydrochloride (20 nM) (B6292-PKC inhibitor), forskolin (50 μM) (F6886-PKA/adenylate cyclase activator), and PMA (80 nM) (P1585-PKC activator) were purchased from Sigma-Aldrich.

### Knockout podocyte cell lines.

PKC-α– and CREB-disrupted podocytes were generated using CRISPR/Cas9 to target the *PRKCA* gene and *CREB1* gene in immortalized human podocytes. A guide RNA targeting the translated region of PRKCA exon 1 (CAACGACTCCACGGCGTCTC) or CREB 1 exon 2 (GGAGCCGAGAACCAGCAGAG) was ligated into the LentiGuide Puro vector (85) and confirmed by Sanger sequencing. Virus was generated by cotransfection of vector, pMDG2, and psPAX2 in HEK293T cells and used to infect a podocyte line that stably expressed Cas9 endonuclease. Cells were selected with puromycin. Successful CRISPR gene editing was confirmed by Sanger sequencing with primers for PRKCA exon 1 coding region (CCDS11664.1, forward, CACCGGGCTGTCAGTGAG, reverse, CGGTTCCAAGTTATCGGAGT) or CREB1 exon 1 (CCDS2374.1, forward, ACCACTGCACCTCTCCTTGT, reverse, TTCTGGATCATTTCACTAAAAAT).

### qPCR.

RNA was isolated using TRIzol/chloroform extraction as described (86) or using the QIAGEN miRNeasy Mini Kit according to the manufacturer’s instructions. Cells were treated for 4 hours and experiments repeated 3 times. Mouse eyes were extracted and the retina separated from the choroid/RPE. From 2 to 3 animals per condition were studied in individual experiments, and these were repeated 3 times. Complementary DNA was made using the High-Capacity RNA to cDNA Kit (Life Technologies) or the QuantiTect Reverse Transcription Kit (QIAGEN). SYBR Green (Sigma-Aldrich) primer sequences are detailed in the Supplemental Table 1 (Life Technologies). SYBR Green reaction mix was used (Sigma-Aldrich) on the StepOnePlus Real-Time PCR system (Applied Biosystems) or the CFX 96 (Bio-Rad). TaqMan qPCR was performed on the Bio-Rad CFX 96 using murine primers (Supplemental Table 2, Life Technologies). Each reaction was run in triplicate. Relative expression was determined compared with GAPDH or actin controls. Changes in mRNA expression in treated cells relative to respective controls were then calculated using the ΔΔCT method.

### ISH.

The Affymetrix QuantiGene View RNA ISH system was used according to the manufacturer’s instructions. Briefly, tissue was dissected and fixed in 4% PFA for 6 hours for eyes and 24 hours for solid organs. Sections were further fixed in 4% formaldehyde at 4°C for 16 hours prior to ISH. Protease solution was applied to eyes (1:25 dilution) for 10 minutes and for solid organs (1:100) for 20 minutes at 40°C. Probes for murine VEGF (VB1-16139 and VB6-12843), murine C3 (VB1-13781), human VEGF (VA1-16136), and human CFH (VA6-16509) were used. Murine CFH was designed and made by Affymetrix using the accession number NM_009888.3 (VB1-16095). Fluorescent detection was used to analyze the ISH with DAPI nuclear costain. Eight mice per condition were analyzed for each experiment. Samples were collected from 3 independent experiments.

### Cytometric bead array.

Human C3a and C4a were examined using the BD Human Anaphylatoxin Cytometric Bead Array (CBA) Kit. Aqueous humor samples were analyzed at 1:5 dilution. Cytokines including VEGF, MIP-1B, MCP1, IL-6, and IL-8 were measured using the BD CBA Flex Immunoassay Kit (BD) as described (87). A list of all other cytokines analyzed using this assay is given in the Supplemental Methods.

### ELISA.

Human C5a was measured in aqueous humor using the Quidel MicroVue C5a EIA (A021) according to the manufacturer’s instructions. Human CFH was measured in conditioned media using an AbCam ELISA (ab137975). Murine CFH was measured using a LifeSpan BioScience ELISA (LS-F4381). Murine C5b-9 was measured using a myBioSource ELISA (MBS703522). All samples were analyzed in duplicate. The plates were imaged at 450 nm and 570 nm to correct for nonspecific signal, using a BioTek Synergy 2 plate reader. Data were analyzed using Gen5 BioTek software.

### Western blotting.

Lysates were made using RIPA lysis buffer with 1% phosphatase and protease inhibitors (Sigma-Aldrich). Protein concentration was measured by BCA assay (Thermo Scientific). Samples were resolved on 10% SDS-PAGE or 4%–12% gradient NuPage Bis-Tris gel (Life Technologies) under reducing conditions and blotted onto polyvinylidene fluoride or nitrocellulose (Immobilon-p, Immobilon-Fl, Nitrocellulose-Fl, Millipore). Membranes were blocked in Licor blocking buffer, 5% BSA, or skim milk. Primary antibodies were incubated overnight at 4°C (Supplemental Table 3). Blots were subsequently washed in TBST and incubated with secondary antibody (Supplemental Table 4). For fluorescent Western blots, the Licor Odyssey Fc was used to detect bands. Alternatively, an ECL chemiluminescence system (Amersham Biotech) was used and images acquired using a ChemiDoc-It imager (UVP). Densitometry was performed using Quantity One software (v4.6.5, Bio-Rad Laboratories) or ImageJ (NIH).

### Meso scale discovery.

Protein concentrations were also measured using meso scale discovery (MSD) technology per the manufacturer’s instructions. Murine samples were analyzed using the V-plex proinflammatory panel 1 kit (K15048D) or the U-plex VEGF assay (K152UVK). Human condition media samples were analyzed using the V-plex human VEGF kit (K151RHD).

### Immunofluorescence.

Cells were grown and differentiated on either glass coverslips or chamber slides in serum-containing media. After treatment, cells were fixed in 4% PFA (Sigma-Aldrich) and blocked in 5% BSA. Fresh-frozen sections were fixed in 4% PFA and blocked in 10% goat or donkey serum with 3% BSA and 0.1% Triton X-100 in PBS. Primary antibodies were incubated overnight at 4°C before 1 hour of incubation with secondary antibodies (Supplemental Table 3 and 4) at room temperature, followed by DAPI nuclear stain. Samples were mounted with Slowfade Gold Anti-Fade Mount (Life Technologies) prior to imaging using the Leica DMI 6000B microscope, Leica confocal imaging spectrophotometer system (TCS-SP2), or the Zeiss LSM 700 or 710. Imaging was carried out at room temperature with a HCX PL FLUOTAR L 40 × 0.6 objective lens with an optical zoom of 40 or 20×/0.8 Dry Plan-Apochromat. Images were taken blindly with only 1 control slide labeled. Microscope settings were determined using a control slide and the brightest staining slide for each experiment. They were then maintained for all remaining comparative slides. Image analysis was carried out using ImageJ (NIH). For all raw images, mean fluorescence intensity (MFI) values were calculated and corrected for cell number determined by DAPI nuclear stain to allow semi-quantitative analysis of immunofluorescence (IF). Eight images per condition were analyzed from 4 independent experiments. For renal tissue, MFI was calculated for individual glomeruli and was corrected for cell number after counting DAPI-stained nuclei. These values were further corrected by subtracting background fluorescence intensity. Analysis incorporated 15 to 20 glomeruli per mouse from 3 different sections. At least 8 mice per condition were analyzed from 4 independent experiments. These studies were based on the use of IF and quantification by others (43, 88, 89). More details on antibodies used are given in Supplemental Table 3 and 4. For retinal sections, the number of positive cells was counted and corrected for retinal length (mm) from 3 different sections per eye.

### Factor H resynthesis assay.

Cells were grown for IF. Once differentiated, they were washed in PBS and treated with 0.1 M acetic acid (Sigma-Aldrich) for 30 minutes at 37°C (90). After washing, cells were treated with VEGF, bevacizumab, ranibizumab, afilbercept, or inhibitors in serum-free media for 24 hours or immediately fixed in 4% PFA. Fixed, untreated cells were also used as controls. CFH cell-surface expression was assessed according to the IF protocol described above. Images were analyzed as described. Ten images were obtained per condition for each experiment, and experiments were repeated 4 times.

### Complement challenge assay.

Cells grown for IF were incubated with 40% rabbit serum for 30 minutes at 37°C to establish cell-surface antigen-antibody complexes and then washed in PBS. The classical and alternative complement pathways were activated by adding 30% human C7 deficient (C7d) serum (Quidel) diluted in gelatin veronal buffer (GVB^2+^, Sigma-Aldrich) for 30 minutes at 37°C. Heat-inactivated normal human serum and GVB buffer alone were used as controls. The absence of protein C7 meant that MAC did not form, and so cells remained intact. Complement activation was indirectly assessed using mouse anti-human antibodies against C3d and C4d (Quidel, Supplemental Table 3). These markers were used to assess complement activation because C3b and C4b are degraded in the presence of serum-based proteases such as factor I (91). These degradation products covalently bind to cells and tissue and persist for hours after complement activation. Their use was previously validated (92). Primary antibodies were applied for 60 minutes at room temperature after PFA fixation followed by secondary antibody (Life Technologies) applied for 1 hour at room temperature. Quantification was performed as for IF using ImageJ (NIH). Ten images per condition were obtained per experiment, and 4 independent repeats were performed.

### Statistics.

Statistical analyses were carried out using PRISM (version 6, GraphPad Software). Experiments involving 2 groups were compared using unpaired, 2-tailed *t* tests except analysis of aqueous humor, for which paired, 2-tailed *t* tests were used. Mann-Whitney *U* test was used to compare time to recurrence data from ARMD patients. Multiple comparisons were made using 1-way ANOVA with Bonferroni’s post hoc analysis. Two-way ANOVA with Bonferroni’s post hoc analysis was used to compare the 2 different genotypes of RPE cells or podocytes treated with VEGF and VEGF antagonists. Pearson’s correlation coefficient was calculated to assess correlation between 2 variables. Results were significant at *P* < 0.05. Variation is expressed as SEM.

### Study approval.

Ethical approval was obtained from the Tokyo Medical University institutional review board for human aqueous humor samples. The Southwest Multicentre Research Ethics Committee granted ethical approval for the creation of patient-derived glomerular cell lines and the National Eye Institute/NIH for iPS cell RPE cell lines. All patients gave informed consent. Animal experiments were approved by the institutional animal care and use committees at Mount Sinai Hospital (Toronto, Canada) and the Scripps Research Institute.

## Author contributions

LSK conceptualized, designed, and performed experiments and wrote and edited the manuscript. AR helped design complement experiments and reviewed and edited the manuscript. RF, LA, DF, EA, and SS performed experiments. GIW, SCS, and RJC gave guidance on glomerular cell experiments. YU and PDW provided advice on retinal experiments and edited the manuscript. YU provided the aqueous humor samples. KRB and JG made the podocyte CRISPR cell lines. VK performed experiments on the podocyte CRISPR cell lines. KB and RS provided the iPS cells differentiated into RPE. IPM made and provided the anti-mouse and human VEGF and edited the manuscript. MAS supervised the renal work and reviewed and edited the manuscript. MF supervised the retinal work and reviewed and edited the manuscript.

## Supplementary Material

Supplemental data
