# Supplementary material for: VEGF regulates local inhibitory complement proteins in the eye and kidney
Source: J Clin Invest. 2016 Dec 5;127(1):199–214. doi: 10.1172/JCI86418 (PMC5199702; doi:10.1172/JCI86418)
Supplement: Supplemental data [file jci-127-86418-s001.pdf]

Supplementary Materials

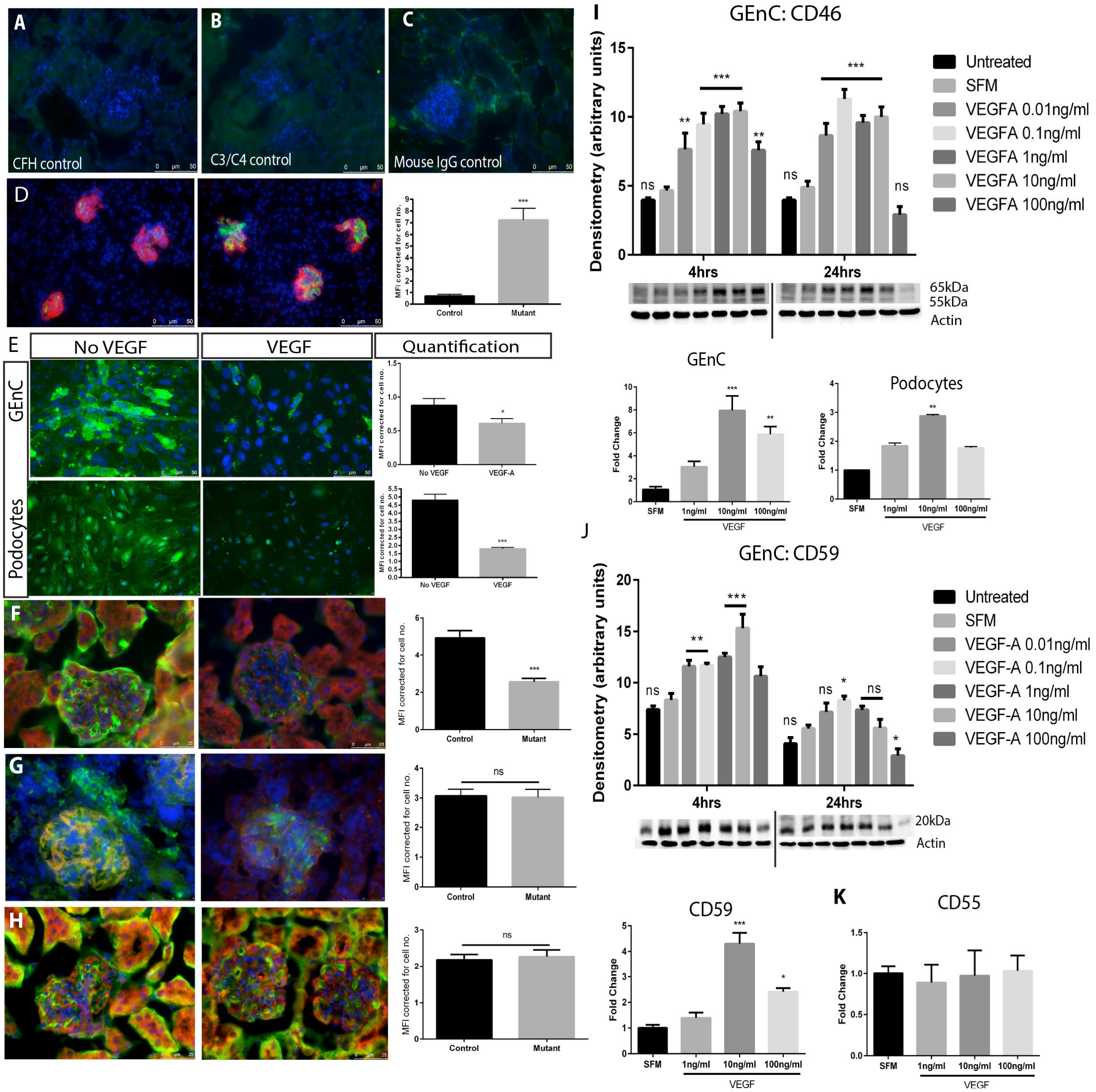

Figure S1: VEGF up regulates glomerular cell expression of complement regulators in a dose dependent manner

Murine tissue was treated with isotype control antibodies to compare with CFH staining (**A**) and C3/C4 staining (**B**). Since the presence of antibodies could influence the complement protein staining patterns seen, podocyte specific Vegf knockout mice were also stained with AlexaFlur 488 labelled murine IgG. No glomerular staining was detected (**C**). These mice did show glomerular staining of C4 that could suggest classical or lectin pathway activation (**D**). Antibody-mediated complement activation on human glomerular endothelial cells (GEnC) and podocytes also resulted in C4d deposits that were reduced by VEGF treatment (**E**). Together this suggested that VEGF could regulate complement proteins outwith the alternative pathway. Considering these findings the podocyte specific Vegf knockout mice were studied for expression of other complement regulators. They showed a reduction in glomerular CD59a staining (**F**) but not CD55 (**G**) or Crry staining (**H**). Human GEnC also showed VEGF dose dependent changes in the membrane regulator CD46 protein after 4 and 24 hours treatment and both GEnC and podocytes showed changes in CD46 RNA after 4 hours of VEGF treatment (**I**). GEnC also showed VEGF dose depend changes in CD59 (**J**) but not CD55 (K). Results shown are representative of four independent experiments. Western blot densitometry was corrected for actin loading. Western blotting and qPCR n=4. Immunofluorescence semi-quantification was performed by measuring mean fluorescence intensity (MFI) which was corrected for cell number. Ten images per condition were obtained for cell culture fluorescent studies during each experiment. Six mice were analysed for each condition during each experiment. * $p<0.05$, ** $p<0.01$, *** $p<0.001$, **** $p<0.0001$.

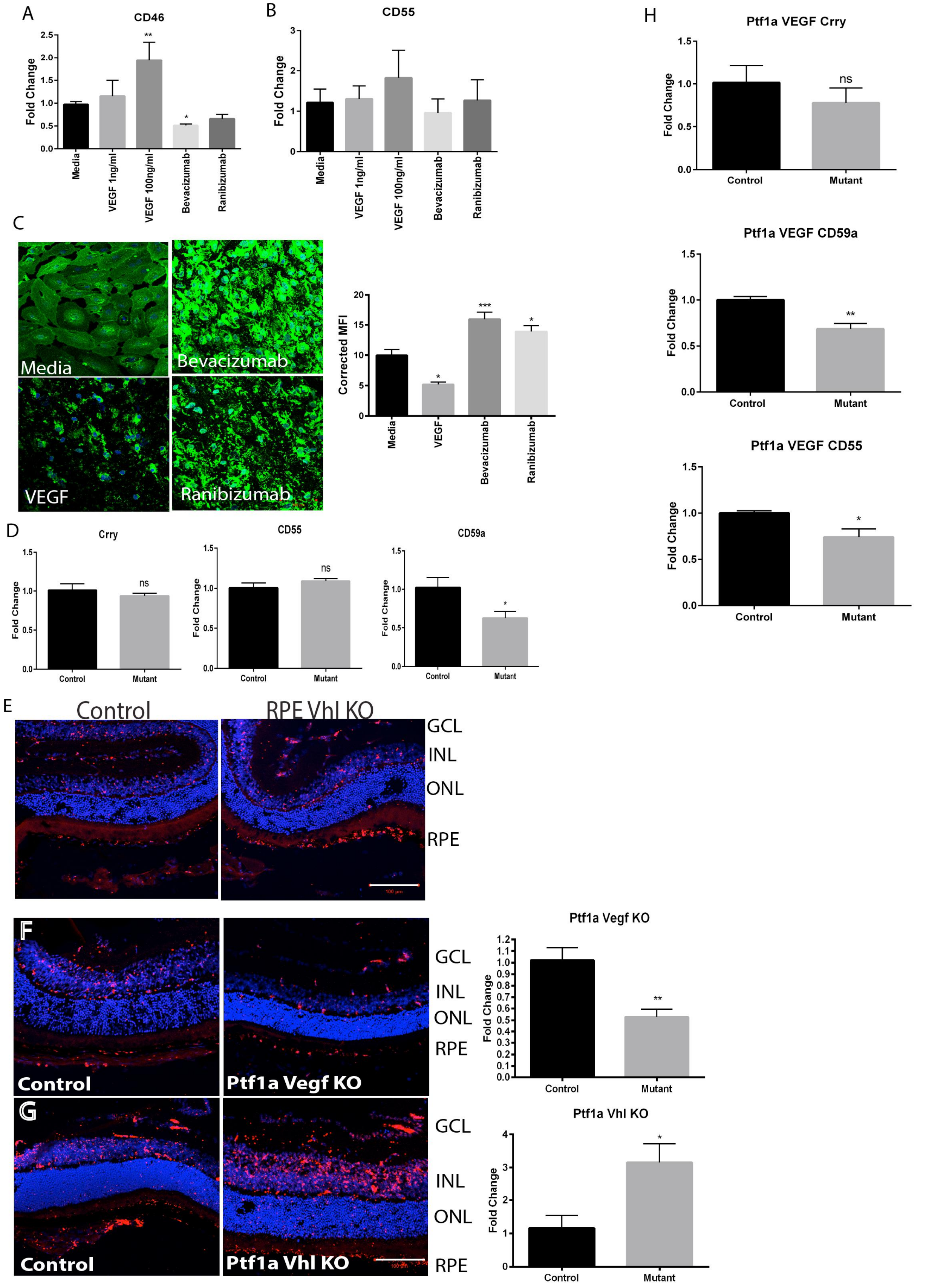
A
CD46
Fold Change
Media
VEGF 1ng/ml
VEGF 100ng/ml
Bevacizumab
Ranibizumab
B
CD55
Fold Change
Media
VEGF 1ng/ml
VEGF 100ng/ml
Bevacizumab
Ranibizumab
C
Media
Bevacizumab
VEGF
Ranibizumab
Corrected MFI
Media
VEGF
Bevacizumab
Ranibizumab
D
Crry
Fold Change
ns
Control
Mutant
CD55
Fold Change
ns
Control
Mutant
CD59a
Fold Change
Control
Mutant
E
Control
RPE Vhl KO
GCL
INL
ONL
RPE
F
Control
Ptf1a Vegf KO
GCL
INL
ONL
RPE
Ptf1a Vegf KO
Fold Change
Control
Mutant
G
Control
Ptf1a Vhl KO
GCL
INL
ONL
RPE
Ptf1a Vhl KO
Fold Change
Control
Mutant
H
Ptf1a VEGF Crry
Fold Change
ns
Control
Mutant
Ptf1a VEGF CD59a
Fold Change
Control
Mutant
Ptf1a VEGF CD55
Fold Change
Control
Mutant

Figure S2: Retinal complement regulators were increased by VEGF treatment and reduced by VEGF inhibition

Human primary RPE cells showed a dose dependent increase in CD46 RNA, which was reduced by VEGF antagonism with bevacizumab or ranibizumab (**A**). No changes in CD55 RNA were detected in these cells (**B**). Pre-treatment of these cells with VEGF reduced C4d deposits when antigen-antibody complexes activated complement but VEGF antagonism increased these deposits (**C**). *In vivo*, inducible RPE-specific Vegfa knockout mice showed less CD59a but not CD55 or crry 3 days post induction (**D**). Conversely, in the RPE specific Vhl knockout mice which have increased Vegf, there was more Cfh (red) RNA seen around the RPE/choriocapillaris three days post induction using *in situ* hybridization(**E**). Amacrine/horizontal cell (Ptf1a Cre) specific Vegf knockout (KO) retinas also showed less CFH transcript by qPCR (**F**) while the Vhl knockout showed significantly more CFH RNA confirming the results obtained by *in situ hybridization* (**G**). These mice also showed reduced CD59a and CD55 but not crry at the RNA level (**H**). Results shown are representative of four independent experiments. Immunofluorescence semi-quantification was performed by measuring mean fluorescence intensity (MFI) which was corrected for cell number. Ten images per condition were obtained for cell culture fluorescent studies during each experiment. For cell experiments n=4. 6-8 mice were used per condition. Statistics: ANOVA * $p<0.05$, ** $p<0.01$, *** $p<0.001$, **** $p<0.0001$.

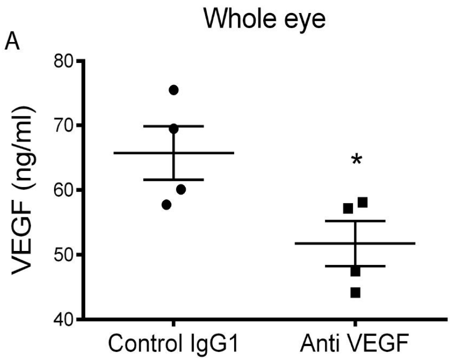

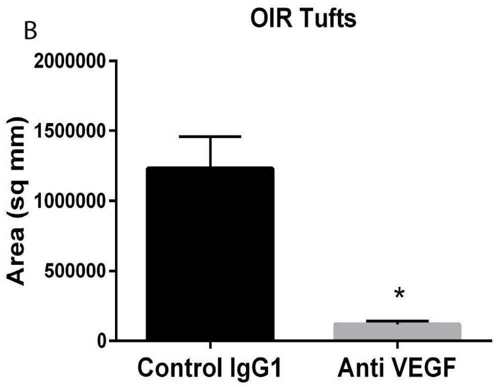

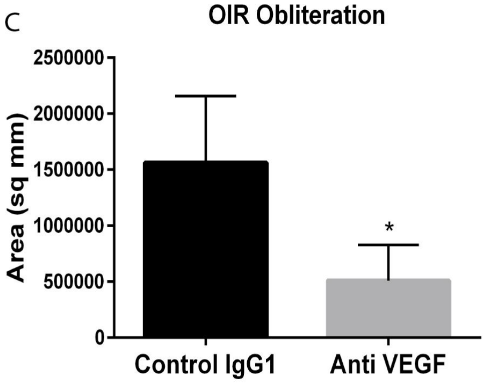

Representative images

Control IgG1

Anti VEGF

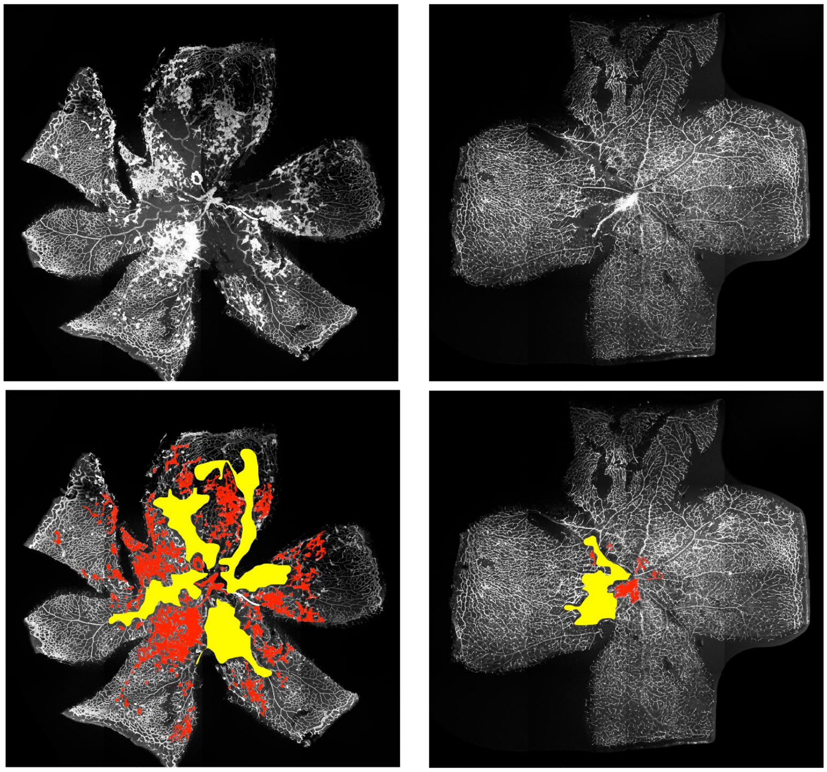

Figure S3: Anti-mouse VEGF reduced neovascular tuft formation in murine oxygen induced retinopathy model

Wild type C57BL6 mice were injected with intravitreal anti-VEGF or control IgG1 and then whole eyes removed for protein analysis of VEGF 48 hours later. There was a significant reduction in VEGF concentration in mice that received anti mouse VEGF (**A,** n=4). C57BL6 mice underwent oxygen-induced retinopathy (OIR) as previously described(1, 2). Anti mouse VEGF or control was injected intravitreally on day 12 when pups were removed from hyperoxia and entered the relative hypoxia stage being housed back in room air. On day 17 eyes were analysed to look at neovascular tuft formation and retinal obliteration. This was quantified using Photoshop. Areas of tufts and obliteration were calculated and compared. Anti-mouse VEGF treatment significantly reduced tuft formation (**B**) and obliteration (**C**). Representative images are shown, tufts are highlights in red, obliteration is highlighted in yellow.*$p<0.05$, n=6, Statistic: Unpaired, two tailed t-test. *$p<0.05$

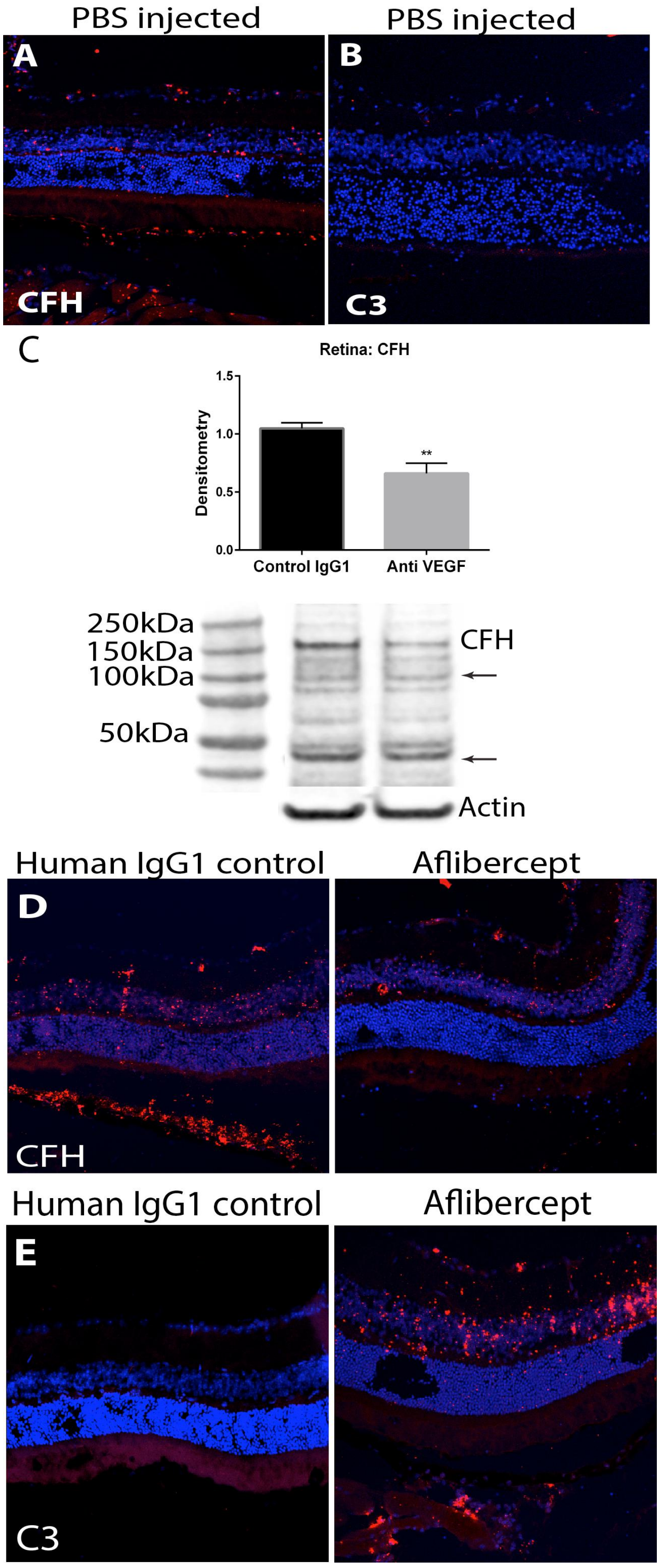

PBS injected
PBS injected
A
B
CFH
C3
C
Retina: CFH
Densitometry
1.5
1.0
0.5
0.0
**
Control IgG1
Anti VEGF
250kDa
150kDa
100kDa
50kDa
CFH
Actin
Human IgG1 control
Aflibercept
D
CFH
Human IgG1 control
Aflibercept
E
C3

Figure S4: Intravitreal anti VEGF injections reduced murine CFH but increased C3

Mice injected with intravitreal PBS showed no changes in CFH (**A**) or C3 (**B**) RNA expression on *in situ* hybridization. Mice injected with anti mouse VEGF showed reduced retinal CFH compared to those injected with control IgG1, confirmed by western blotting (**C**). There were extra bands seen in the retinal lystaes (shown by arrows). These were not seen in the choroid/RPE samples and were not removed by different blotting or blocking techniques. They may represent expression of murine complement factor H related proteins (100kDa, 38kDa). Mice were also injected with intravitreal aflibercept (2mg/ml) or control human IgG1. There was a reduction in CFH transcript (**D**) and increase in C3 transcript (**E**) similar to the results obtained from anti mouse VEGF injections. In situ hybridization images are representative of 8 replicates. Western blotting, n=4. Statistics: unpaired, two tailed t-test. ** $P<0.01$.

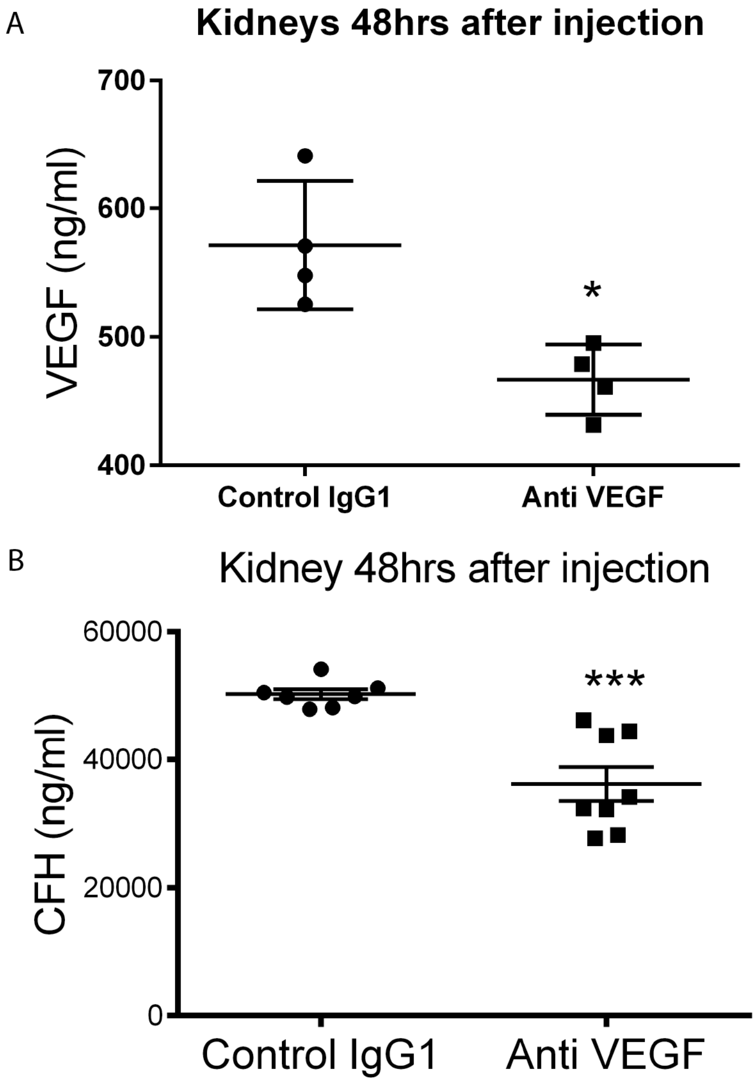
A
Kidneys 48hrs after injection
VEGF (ng/ml)
700
600
500
400
*
Control IgG1
Anti VEGF
B
Kidney 48hrs after injection
CFH (ng/ml)
60000
40000
20000
0
***
Control IgG1
Anti VEGF

Figure S5: Kidneys from mice injected with intravitreal anti VEGF showed reduced VEGF and CFH 48 hours later

Whole kidneys were analyzed from mice 48 hours after they had been injected with intravitreal anti VEGF or control IgG1. VEGF concentration was significantly reduced in those who received anti mouse VEGF (n=4, *$p<0.05$). CFH concentration was also significantly reduced in the kidneys of these mice (n=8, ***$p<0.001$). Statistics: unpaired, two tailed t-test.

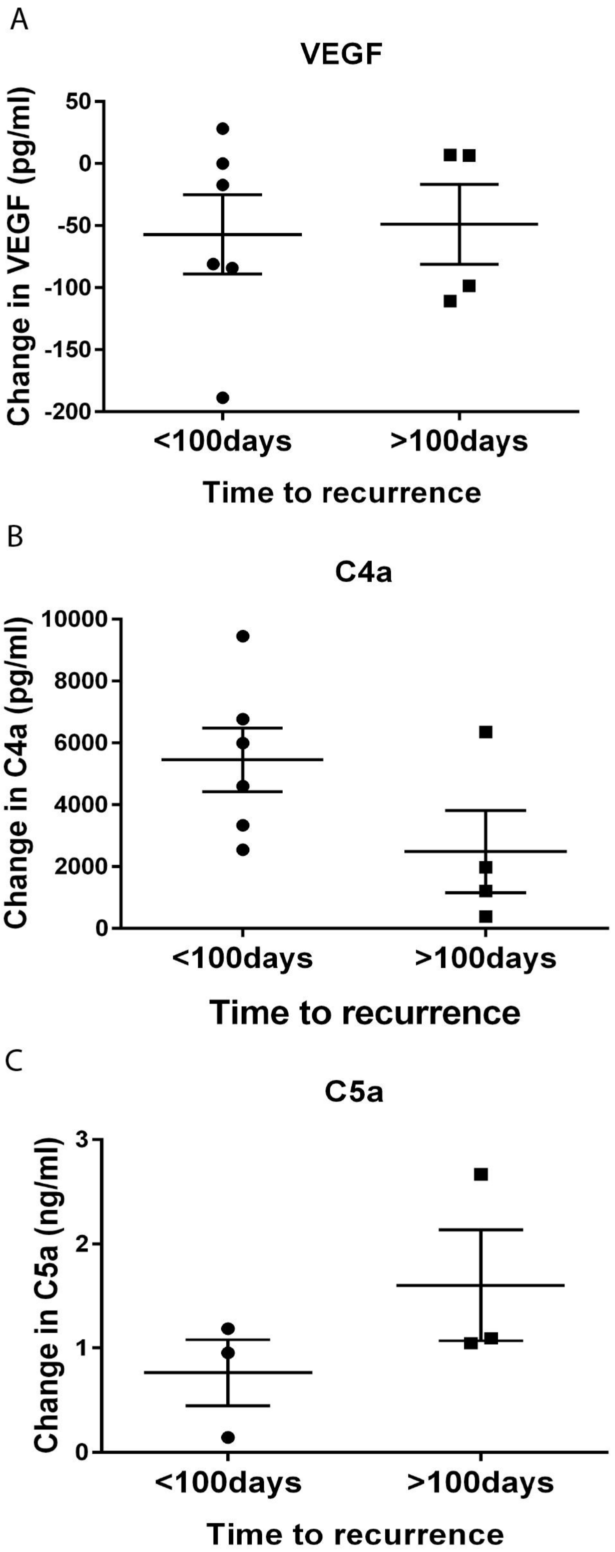
A
VEGF
Change in VEGF (pg/ml)
50
0
-50
-100
-150
-200
<100days
>100days
Time to recurrence
B
C4a
Change in C4a (pg/ml)
10000
8000
6000
4000
2000
0
<100days
>100days
Time to recurrence
C
C5a
Change in C5a (ng/ml)
3
2
1
0
<100days
>100days
Time to recurrence

Figure S6: No differences in VEGF, C4a or C5a correlated with time to recurrence in aqueous humour from AMD patients treated with intravitreal bevacizumab.

Aqueous humour samples from 10 AMD patient eyes obtained before and 48 hours after intravitreal bevacizumab. The concentration change of VEGF, C4a and C5a were measured in these samples. This was then compared between those patient who relapsed before 100days of treatment and those who relapsed more than 100 days after. There were no significant changes between these groups. n=3-6. Statistics: unpaired, two tailed t-test.

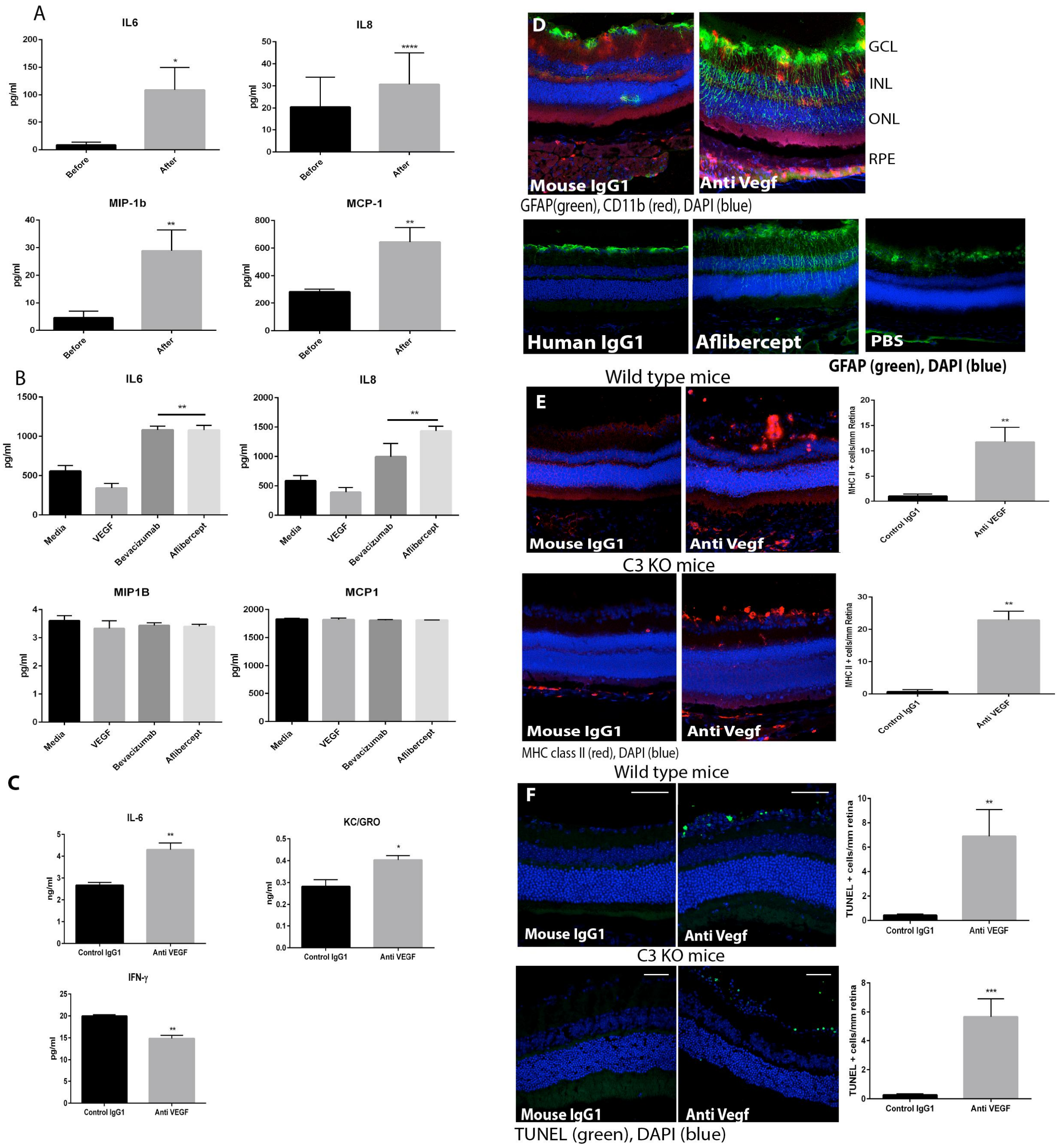

A
IL6
IL8
MIP-1b
MCP-1
pg/ml
Before
After
B
IL6
IL8
MIP1B
MCP1
Media
VEGF
Bevacizumab
Aflibercept
C
IL-6
KC/GRO
IFN-γ
ng/ml
Control IgG1
Anti VEGF
D
GCL
INL
ONL
RPE
Mouse IgG1
Anti Vegf
GFAP(green), CD11b (red), DAPI (blue)
Human IgG1
Aflibercept
PBS
GFAP (green), DAPI (blue)
Wild type mice
E
MHC II + cells/mm Retina
C3 KO mice
MHC class II (red), DAPI (blue)
Wild type mice
F
TUNEL + cells/mm retina
C3 KO mice
TUNEL (green), DAPI (blue)

Figure S7: Intravitreal injection of anti VEGF agents causes inflammation and pro-inflammatory cytokine production in the retina

Aqueous humour was obtained from 10 ARMD patient eyes before and then 48 hours after a single intravitreal bevacizumab. The samples taken after bevacizumab showed increased levels of proinflammatory cytokines including interleukin-6 (IL-6), interleukin-8 (IL-8), monocyte chemoattractant protein 1 (MCP-1) and macrophage inhibitory protein -1β (MIP-1β) (**A,** n=10). Human retinal pigment epithelial (RPE) cells in vitro also produced increased IL-6 and IL-8 24 hours after VEGF antagonism with bevacizumab or aflibercept (**B,** n=4). MCP-1 and MIP-1β were detected in the RPE condition media but were not changed with VEGF antagonism. Murine retinas from wild type mice analysed 48 hours after a single dose of intravitrel anti-mouse VEGF also showed increased IL-6 and K/GRO which is the mouse equivalent of human IL-8 (**C,** n=6). Wild type mice injected with anti-VEGF also showed positive Muller glia staining for glial fibrillary acidic protein (GFAP-green) (**D,** n=8) as did mice injected with intravitreal aflibercept but not those injected with PBS or IgG controls. There was also evidence of microglia activating with MHC class II expression (**E,** n=8, red). These are features of activation of both these cell types which is suggestive of retinal inflammation. Comparable numbers of MHC class II positive cells were found in C3 knockout animals compared to controls (**E)**. There were also more apoptotic cells found in the retinas of mice injected with anti-VEGF as shown by TUNEL staining (**F,** n=8). This predominantly affected the ganglion cell layer. Similar numbers of TUNEL positive cells were found in C3 knockout animals tested at the same time point (**F**). Immunofluorescent images are representative of 8 replicates. A, C,E,F: unpaired two tailed t-test. B: ANOVA. * p<0.05, ** p<0.01, *** p<0.001, **** p<0.0001.

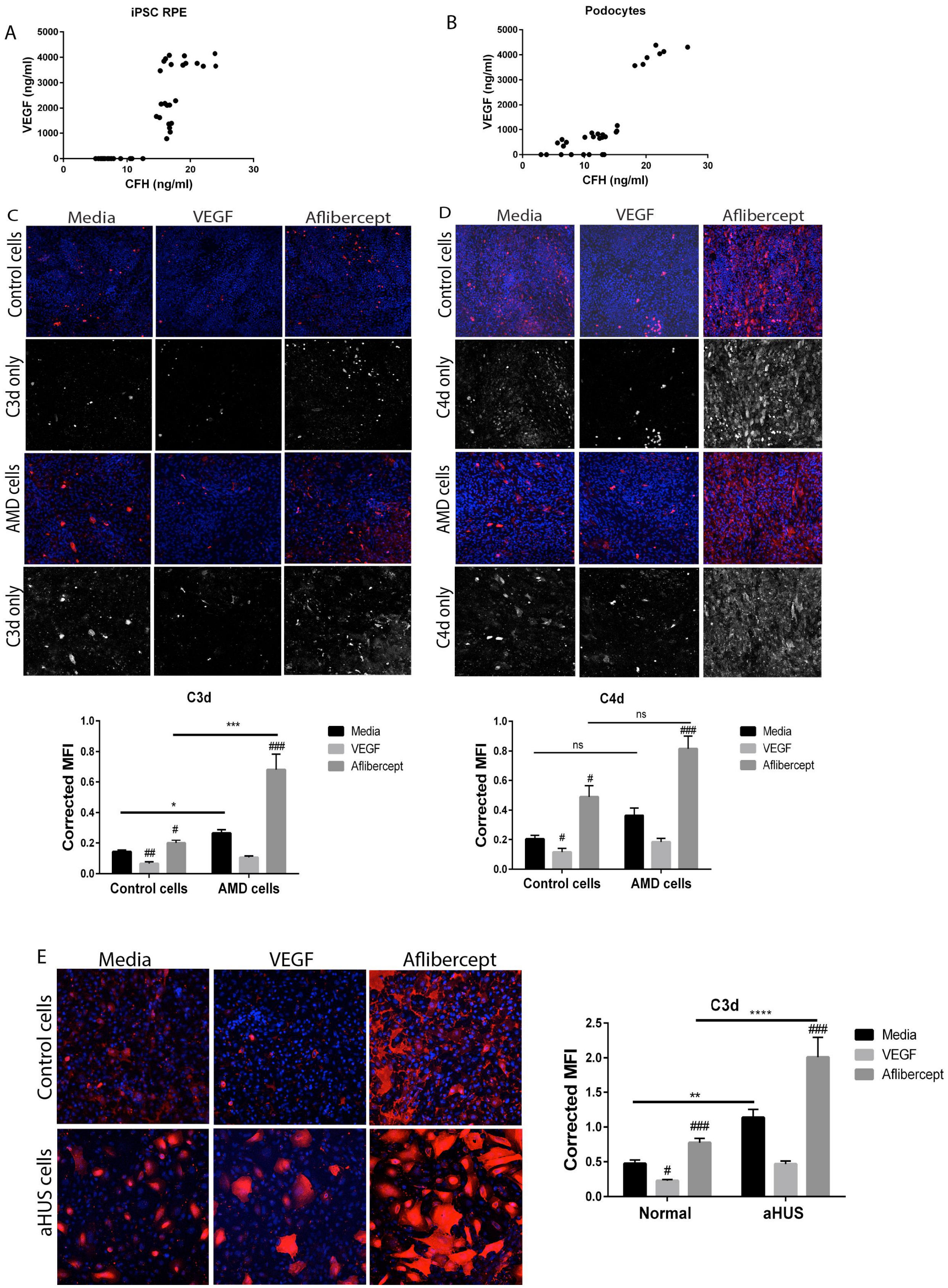

A
iPSC RPE
VEGF (ng/ml)
CFH (ng/ml)
B
Podocytes
VEGF (ng/ml)
CFH (ng/ml)
C
Media
VEGF
Aflibercept
Control cells
C3d only
AMD cells
C3d only
D
Media
VEGF
Aflibercept
Control cells
C4d only
AMD cells
C4d only
C3d
Corrected MFI
Control cells
AMD cells
Media
VEGF
Aflibercept
C4d
Corrected MFI
Control cells
AMD cells
ns
E
Media
VEGF
Aflibercept
Control cells
aHUS cells
C3d
Corrected MFI
Normal
aHUS

Figure S8: Human cells with disease associated CFH genetic variants showed increased complement activation after aflibercept treatment

Human induced pluripotent stem cell derived RPE (iPSC RPE) with or without the ARMD 402H CFH polymorphism were treated with VEGF or bevacizumab or aliberecept. VEGF concentration in the cell culture supernatant was then measured and correlated with CFH concentration. A significant correlation was found with a Pearson correlation co-efficient of 0.86 (95% CI 0.75-0.92, p<0.0001) (**A,** n=30). Human podocytes carrying with a normal CFH allele or an atypical HUS associated mutation in CFH were treated in the same way. The correlation between VEGF and CFH in these cells was 0.87 (95% CI 0.75-0.93, p<0.0001) (**B-**n=30). The iPSC RPE and podocytes were then exposed to complement activation in vitro after VEGF or aflibercept treatment. Without treatment the ARMD associated RPE showed more C3d when compared to the control cell line. This effect was increased with bevacizumab treatment (**C**-black lines show comparisons, * p<0.05, *** p<0.001). When individual cell lines were compared, RPE cells treated with the VEGF antagonists aflibercept showed more cell surface C3d after complement activation than those treated with VEGF or media alone (**C**-# p<0.05, ## p<0.01, ### p<0.001). There was also more C4d on RPE treated with aflibercept compared to cells treated with VEGF or media alone (**D**--# p<0.05, ### p<0.001). However, both cells lines showed equal C4d staining showing comparable classical pathway activation (**D-**black lines show comparison, ns=not significant). Complement depositions shown in red (**C** C3d red, DAPI blue, **D** C4d red, DAPI blue). Lower panels show only complement deposits shown in white. Human podocytes treated with aflibercept also showed similar results with increased C3d (red, DAPI blue) detected in podocytes derived from an atypical HUS (aHUS) patient after aflibercept treatment compared to normal control cells (**E-**black lines show comparison between cell lines, ***p<0.001). VEGF treatment reduced these deposits in control cells (**E**, # p<0.05). Representative images are shown from four independent experiments. 10 images were obtained for each condition per individual experiment. Quantification of fluorescence was measured as mean fluorescence intensity (MFI) and was corrected for cell number. Two-way ANOVA was performed to compare results from two different genotypes of RPE cells or podocyte. Statistics comparing the *same* cell types exposed to different treatments are denoted as # p<0.05, ## p<0.01, ### p<0.001. Statistics comparing *different*

cell types are denoted as ns= not significant, * p<0.05, ** p<0.01, *** p<0.001, **** p<0.0001. Black bars show which conditions were compared.

Table 1: Quantitative PCR primers used with Sybr Green

Primer name	Sequence
Human CFH forward	CCT GAT CGC AAG AAA GAC CAG
Human CFH reverse	ACT GAA CGG AAT TAG GTC CAA C
Human GAPDH forward	TGT GGG CAT CAA TGG ATT TGG
Human GAPDH reverse	ACA CCA TGT ATT CCG GGT CAA T
Human CD46 forward	ATA CAT GGC TAC CTG TCT CAG AT
Human CD46 reverse	CCA AAC TCG TAA GTC CCA TTT GC
Human CD55 forward	AGG CCG TAC AAG TTT TCC CG
Human CD55 reverse	CCT TCT CGC CAG GAA TTT TCA C
Human CD59 forward	TTT TGA TGC GTG TCT CAT TAC CA
Human CD59 reverse	ATT TTC CCT CAA GCG GGT TGT
Mouse Cfh forward	AGGCTCGTGGTCAGAACAAC
Mouse Cfh reverse	GTTAGACGCCACCCATTTTCC
Mouse Crry forward	ATGGAGGTCTCTTCTCGGAGT
Mouse Crry reverse	GGCCGAAGGCTACAAGGAG
Mouse CD55 forward	ACCTCCACTCCCAGGAAAAG
Mouse CD55 reverse	TAGAGGAGACACCGACTAGCC
Mouse CD59a forward	GTTAGCCTCACATGCTACCAC
Mouse CD59a reverse	AGGAGAGCAAGTGCTGTTCATA
Mouse beta actin forward	GGCTGTATTCCCCTCCATCG
Mouse beta actin reverse	CCAGTTGGTAACAATGCCATGT

Table 2: Quantitative PCR primers used with Taqman System

Taqman Primer name	Life technology catalog number
Cfh	Mm01299248_m1
C3	Mm01232779_m1
Lox-1/orl1	Mm00454588_g1
P-selectin	Mm01295931_m1
Serpine-1/PAI-1	Mm00435858_m1

Table 3: Primary antibodies

Antibody	Manufacturer	Immunofluorescence concentration	Western blot concentration
Complement factor H	EMD Millipore Calbiochem #341276	1 in 200	1 in 5000
C3d	Quidel #A207	1 in 100	
C4d	Quidel #A213	1 in100	
Mouse C3-FITC	MP biochemical #0855500	1 in 500	
Mouse C4	AbCam #ab11863	1 in 200	
Mouse CFH	Hycult # HM1119	1 in 100	
Podocin	Sigma #P0372	1 in 500	
Nephrin	Acris #BP5030	1 in 500	
Phospho-CREB	Cell signaling #9198		1 in 1000
Total CREB	Cell signaling #9197		1 in 1000
Phospho-CREB	AbCam #ab31387		1 in 1000
Beta Actin	Sigma #A1978		1 in 10,000

Quidel C3d and C4d used as per(3)**.**

Extra antibodies in supplement

Antibody	Source	Immunofluorescence concentration	Western blot concentration
Mouse Cd59a	Gift from Prof Paul Morgan (see ref A below)	1 in 50	
Mouse Crry	Gift from Prof Paul Morgan (see ref B below)	1 in 50	
Mouse CD55	Hycult biotech, clone 3D5, #HM1115	1 in 100	
Human CD46	AbCam		1 in 5000
Human CD59	Millipore		1 in 2500
Mouse GFAP	AbCam # ab7260	1 in 1000	
Mouse CD11b	AbCam # ab8879	1 in 200	
Mouse MHC class II (Phycoeryhtrin)	AbCam # ab93560	1 in 500	

Mouse CD59a and crry antibodies were a gift from Professor Paul Morgan (University of Cardiff, Cardiff, UK).

Reference A for Cd59a antibody: Harris, C. L., S. M. Hanna, M. Mizuno, D. S. Holt, K. J. Marchbank, B. P. Morgan. 2003. Characterization of the mouse analogues of CD59 using novel monoclonal antibodies: tissue distribution and functional comparison. *Immunology* **109**: 117-126.

Reference B for crry antibody: Mizuno, M., C. L. Harris, B. P. Morgan. 2006. Spermatogenic cells distal to the blood-testis barrier in rats lack C3 convertase regulators and may be at risk of complement-mediated injury. *J. Reprod. Immunol.* **69**: 23-34.

Table 4: Secondary antibodies

Antibody	Manufacturer	Immunofluorescence concentration	Western blotting concentration
Anti-goat 594	Life technologies #A11058	1 in 500	
Anti-mouse 647	Life technologies #A31571	1 in 200	
Anti-rat 488	Life technologies #A21208	1 in 500	
Anti-mouse 488	Life technologies #21202	1 in 500	
Anti-rabbit 594	Life technologies #R37119	1 in 500	
Anti-guinea pig 594	Life technologies #A11076	1 in 500	
IRDye 800 anti-goat	Licor #926 32214		1 in 5000
IRDye 800 anti-rabbit	Licor #926 232213		1 in 5000
IRDye 680 anti-mouse	Licor #926 68072		1 in 10000

Supplementary Methods

Oxygen induced retinopathy

The oxygen induced retinopathy model of retinal neovascularization was performed as previously described(1, 2). When pups were removed from hyperoxia, murine anti VEGF or IgG1 control was injected intravireally on day 12. Retinal vasculature was then assessed at day 17 by staining retinal flat mounts with isolectin GS-B4 Alexa fluor 594 (ThermoFischer Scientific, # I21413). Adobe Photoshop was used to quantify neovacular tuft formation and areas of obliteration (expressed as area). Anti VEGF and control injected mice were compared using au unpaired, two tailed T-test. There were 8 mice per group from two independent experiments.

Cytokines measured

The cytokines measured using the BD cytometric bead array flex immunoassay kit were interleukin 1B, 2, 3, 5, 6, 8, 9, 10, IL-12p70, angiogenin, basic FGF, eotaxin, G-CSF, MIP-1A, MIP-1B, MIG, Fas ligand, IP-10, VEGF, RANTES, MCP-1, TNF alpha and interferon-gamma. Only those that showed significant changes before and 48 hours after single intravitreal injection of bevacizumab were included in the main manuscript.

TUNEL staining

Roche Applied Sciences in situ cell death detection kit (#11684795910) was used according to manufacturer instructions. Sections were imaged and quantified as described above. Six different eyes per condition were analyzed from two independent experiments.

References:

1. Scott A, and Fruttiger M. Oxygen-induced retinopathy: a model for vascular pathology in the retina. *Eye (Lond).* 2010;24(3):416-21.
2. Smith LE, Wesolowski E, McLellan A, Kostyk SK, D'Amato R, Sullivan R, and D'Amore PA. Oxygen-induced retinopathy in the mouse. *Invest Ophthalmol Vis Sci.* 1994;35(1):101-11.
3. Barilla-LaBarca ML, Liszewski MK, Lambris JD, Hourcade D, and Atkinson JP. Role of membrane cofactor protein (CD46) in regulation of C4b and C3b deposited on cells. *J Immunol.* 2002;168(12):6298-304.
